# An exploration of prenatal breastfeeding self-efficacy: a scoping review

**DOI:** 10.1186/s12966-024-01641-3

**Published:** 2024-09-02

**Authors:** Liz M. McGovern, Laura O’Toole, Rachel A. Laws, Timothy C. Skinner, Fionnuala M. McAuliffe, Sharleen L. O’Reilly

**Affiliations:** 1 UCD School of Agriculture and Food Science, University College Dublin, Belfield, Dublin 4, D04 V1W8 Ireland; 2 UCD Perinatal Research Centre, School of Medicine, University College Dublin, National Maternity Hospital, Dublin 2, D02 YH21 Ireland; 3 UCD School of Nursing, Midwifery and Health Systems, University College Dublin, Belfield, Dublin 4, D04 V1W8 Ireland; 4 School of Exercise & Nutrition Sciences, Institute for Physical Activity and Nutrition, Deakin University, Geelong, VIC 3220 Australia; 5 Institute of Psychology, University of Copenhagen, Copenhagen K, 1353 Denmark

**Keywords:** Breastfeeding, Lactation, Prenatal care, Antenatal, Self efficacy, Confidence

## Abstract

**Background:**

Breastfeeding self-efficacy is a woman’s self-belief and confidence in her perceived ability to breastfeed. This modifiable determinant is strongly associated with breastfeeding initiation, exclusivity, and duration. It is unclear how important the timing of breastfeeding self-efficacy measurement and interventions are. The prenatal period appears underexplored in the literature and yet a prenatal focus provides increased opportunity for breastfeeding self-efficacy enhancement and further potential improvement in breastfeeding outcomes. This scoping review aims to synthesise the evidence on prenatal breastfeeding self-efficacy, describing for the first time the theoretical frameworks, measurement tools, and interventions used in the prenatal period.

**Methods:**

8 databases were searched using the PCC framework (Problem: breastfeeding, Concept: self-efficacy, Context: prenatal period). From 4,667 citations and 156 additional sources identified through grey literature and snowballing, data were extracted from 184 studies and 2 guidance documents. All were summarised descriptively and narratively.

**Results:**

Just over half (57%) of included studies stated their theoretical underpinning, with Bandura’s Self-Efficacy Theory / Dennis’ Breastfeeding Self-Efficacy Framework predominant. Only half of intervention studies incorporated theory in their design. More intervention studies were undertaken in the past decade than previously, but the level of theoretical underpinning has not improved. Prenatal interventions incorporating theory-led design and using components addressing the breadth of theory, more frequently reported improving breastfeeding self-efficacy and breastfeeding outcomes than those not theory-led. Intervention components used less frequently were vicarious or kinaesthetic learning (52.5%) and involvement of social circle support (26%). The Breastfeeding Self-Efficacy Scales were the most common measurement tool, despite being designed for postpartum use. Overall, issues were identified with the late prenatal timing of breastfeeding self-efficacy investigation and the design, content and phraseology of measurements and interventions used in the prenatal period.

**Conclusion:**

This review provides novel insights for consideration in the design and conduct of breastfeeding self-efficacy studies in the prenatal period. Future research should aim to be theory-led, commence earlier in pregnancy, and embed the breadth of self-efficacy theory into the design of interventions and measurement tools. This would provide more robust data on prenatal breastfeeding self-efficacy’s role in impacting breastfeeding outcomes.

**Supplementary Information:**

The online version contains supplementary material available at 10.1186/s12966-024-01641-3.

## Background

The World Health Organisation (WHO) recommends breastfeeding exclusively for six months and alongside nutritious, complementary foods for two years and beyond [1, 2]. Global breastfeeding rates fall far short of this, with only 44% of the world’s children exclusively breastfed to six months between 2015 and 2020 [3]. Various factors impact decisions around breastfeeding, many of which can be influenced such as type of birth, knowledge of the benefits of breastfeeding, attitudes to infant feeding, breastfeeding self-efficacy, previous breastfeeding experience, and receiving social and professional support [4–8]. Breastfeeding self-efficacy has been a focus of investigation since 1999, when Dennis applied Bandura’s self-efficacy theory [9] to breastfeeding, describing it as the self-belief and confidence a woman has in her perceived ability to breastfeed [10]. Breastfeeding self-efficacy is a construct that can be enhanced over time, as with the perception of competence or confidence in carrying out any task. While breastfeeding knowledge, attitudes and intention also give an indication of breastfeeding confidence and are associated with improved breastfeeding outcomes [5, 7, 11, 12], breastfeeding self-efficacy is modifiable, strongly associated with outcomes, and has received substantial attention in the literature. Those with higher levels have better rates of initiation, duration and exclusivity [13–17], and interventions aiming to improve breastfeeding self-efficacy report having a positive impact on both breastfeeding self-efficacy levels and subsequent breastfeeding outcomes [18–24]. It is critical to focus on maximising this modifiable determinant, especially in countries and cultures with relatively low breastfeeding rates.

The optimal time point for breastfeeding self-efficacy measurement and intervention within pregnancy and postpartum is unclear. The prenatal period appears underexplored in the literature and yet focusing on it prenatally creates more opportunity for its enhancement and further improvement of breastfeeding outcomes. Breastfeeding self-efficacy measurement is used to predict breastfeeding outcomes [25–27], identify those at risk of early cessation [16, 28–30], and evaluate the impact of interventions designed to improve it [31–34]. The early identification of those with low breastfeeding self-efficacy is proposed as a way to maximise the potential amount of time available to improve it through tailored interventions. The benefit of focusing on breastfeeding self-efficacy in early pregnancy has been highlighted [31, 35–38], but some systematic breastfeeding self-efficacy intervention reviews indicate late pregnancy or postpartum are preferred [39–41]. The Breastfeeding Self-Efficacy Scale (BSES) [42] and its shortened form (BSES-SF) [43] are popular postpartum breastfeeding self-efficacy measurement tools. The extent of their use and timing prenatally is unclear, as is the availability and use of prenatally designed breastfeeding self-efficacy tools.

Incorporating theory into breastfeeding self-efficacy intervention design and measurement is recommended [24, 44–46] as a result of the increasing recognition being afforded to the specific design considerations required for complex behaviour change [47]. Self-efficacy theory contains four sources of information that influence a person’s behaviour, effort expended, persistence, thought patterns and emotional reactions, which are: mastery / performance accomplishments, vicarious experience, verbal persuasion, and emotional / physiological states [9, 10]. The incorporation of breastfeeding self-efficacy or related theories into studies conducted in the prenatal period is unknown, along with the extent to which theory is incorporated into measurement tool or intervention design.

A comprehensive knowledge synthesis on prenatal breastfeeding self-efficacy is lacking. This scoping review aims to explore and synthesise the current evidence base and identify the theoretical frameworks, measurement tools, and interventions used in the prenatal period, and their impact on breastfeeding outcomes.

## Methods

The review was conducted in accordance with the Joanna Briggs Institute (JBI) approach for the conduct of scoping reviews, which was informed by Arksey & O’Malley [48] and Levac et al. [49]. The Preferred Reporting Items for Systematic Reviews and Meta-Analysis extension for Scoping Reviews (PRISMA-ScR) [50] guided the design, review, and reporting of this review. The protocol was registered (10.17605/OSF.IO/2JCK7) and previously reported [51].

### Identification of relevant studies and search strategies

The database and grey literature searches covered any published materials from database inception to the date of the search. The databases searched were Medline (Ovid), CINAHL, EMBASE, PsycINFO, Global Health, International Bibliography of Social Sciences (IBSS), Applied Social Science Index and Abstracts (ASSIA) and Web of Science. A variety of grey literature sources were searched: website searches of international organisations with policy, advisory or guidance roles in breastfeeding, including WHO, United Nations International Children’s Emergency Fund (UNICEF), the Global Breastfeeding Collective, the Academy of Breastfeeding Medicine, and La Leche League International, alongside a focused Google search. Theses and dissertations were considered for inclusion from a ProQuest Dissertation & Theses Global database search.

Additional citations for screening were sourced through backward (screening reference lists of included studies), and forward snowballing (identifying new papers citing included studies). Due to the volume of citations within the database search, only studies that used self-efficacy measurement tools designed for use in the prenatal period or a prenatal intervention based on self-efficacy theory were included in the snowballing process. Authors of primary studies were contacted for access to full text articles, when unavailable.

### Study selection

Eligibility criteria followed the PCC framework (Problem: breastfeeding, Concept: self-efficacy, Context: prenatal period) [51]. The citations identified were de-duplicated and screened using Covidence (Veritas Health Innovation, Melbourne, Australia). Screening consistency pilot tests were conducted at both screening stages, with a minimum of 90% agreement between reviewers before proceeding. Title and abstract screening, followed by full text retrieval and screening, were independently conducted by two reviewers (LMcG, LOT). Any disagreements between reviewers were resolved through discussion, or with an additional, independent reviewer (SOR).

Studies were included if they were in English and contained either the prenatal measurement of breastfeeding self-efficacy or breastfeeding confidence, or the prenatal delivery of an intervention explicitly designed to improve breastfeeding self-efficacy. Studies with a postpartum measurement or intervention were also included if they fulfilled the criteria of having a prenatal element. All publication types were considered valid including posters, abstracts, conference papers, protocols, and dissertations, but were excluded when they provided the same content as a corresponding published paper. Dissertations providing additional content and analysis to a published paper were included. An assessment of the quality of evidence within the review was not conducted, as it is beyond the remit of a scoping review.

### Data extraction, analysis and charting

Data were extracted using standard systematic review procedures and conducted by the first author for consistency and due to the large volume of studies included in the review (study design, population / sample, country, aim, self-efficacy measurement and timeframe, other assessment tools, intervention details and timeframe, theoretical framework applied and where, outcome measures, findings, and conclusions). Extraction accuracy was checked independently and at random for a minimum of 20% of studies from the database search by another reviewer (LOT). The studies included in systematic or other reviews were screened against the inclusion criteria and included where relevant. Microsoft Excel was used to collate and analyse the data extracted.

Studies were categorised as intervention studies when they delivered an intervention and presented findings on either breastfeeding constructs (self-efficacy, knowledge, attitudes) or outcomes (duration or exclusivity). Intervention design studies reporting findings only on the acceptability of design aspects were categorised as descriptive studies. The categorisation of theory use was based on each authors’ explicit identification and discussion of theories that underpinned their examination of breastfeeding self-efficacy, either in their choice of measurement tool or their intervention design. Reference to self-efficacy theory solely in a literature review or study introduction was deemed insufficient. The congruence of theory with intervention design was categorised according to each authors’ description of the theory used and linkages made to the components of their intervention. No additional analysis of intervention outcomes was conducted, studies were grouped based on the reported statistical significance of outcomes.

Intervention content was pragmatically grouped into component categories (education, encouragement / support, engagement, vicarious / kinaesthetic learning, involvement / enhancement of social circle). The component categories were broadly mapped to self-efficacy theory antecedents for study outcome comparison, based on perceived potential of intervention elements to influence antecedents.

## Results

**Fig. 1:**
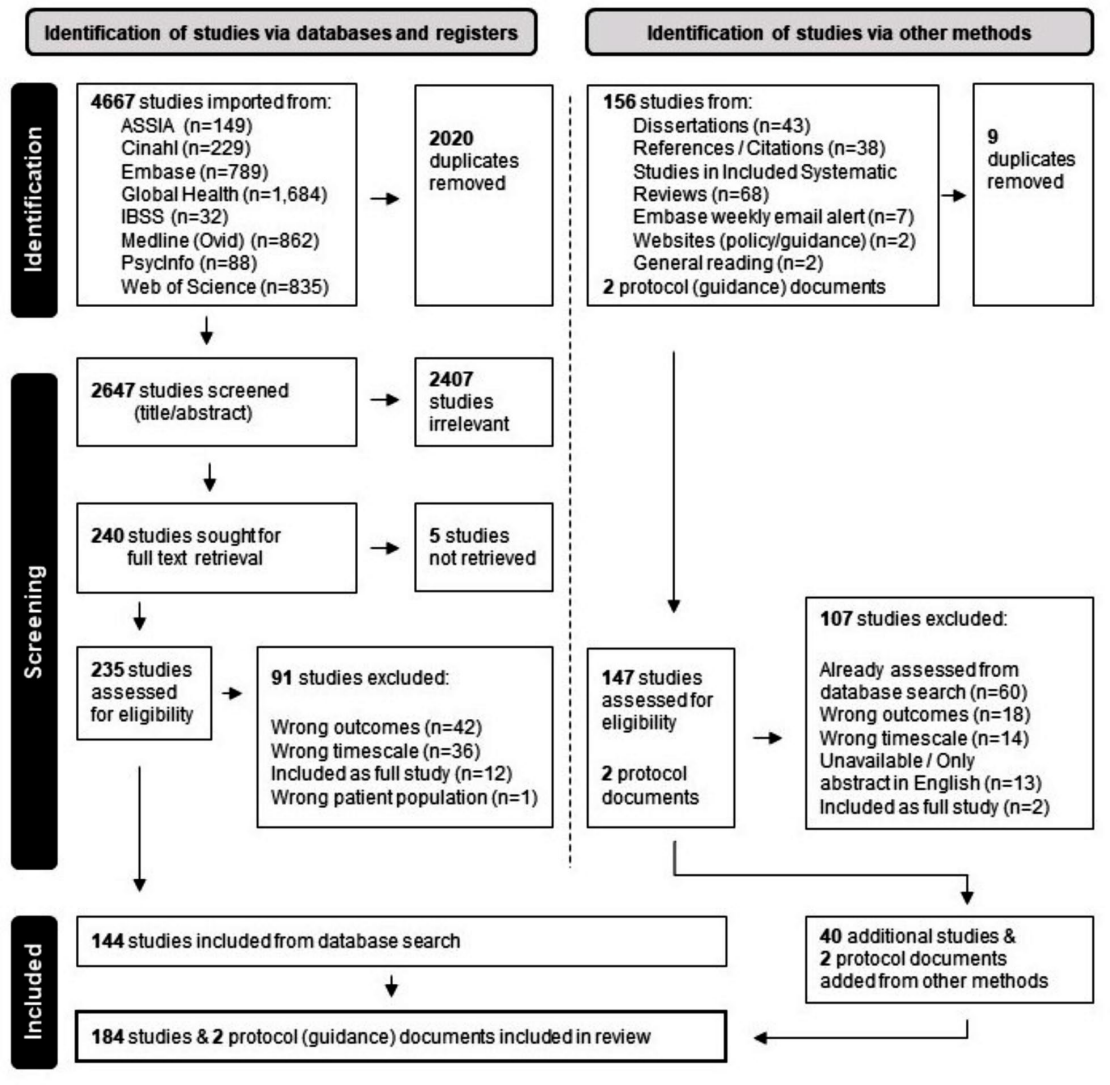
PRISMA flow chart of study inclusion

There were 4,667 studies identified through the database search conducted on September 21st, 2022. After de-duplication, 2,647 studies were title/abstract screened, leading to full text screening of 240 studies. There were 144 studies from the database search that met the inclusion criteria. Additionally, 156 records were identified from other sources after a title/abstract review, including two guidance documents from the Academy of Breastfeeding Medicine [52]. After duplicate removal, 147 studies were screened by the primary author for inclusion, initially against the studies already identified in the database search and then by full text retrieval and screening. There were 40 additional studies added resulting in 184 studies and two guidance documents included in the review. The PRISMA flow chart in Fig. 1 presents further details of the sourcing, screening, and inclusion process. The characteristics of all 184 included studies are presented and referenced in Supplementary Material Table 1.

There were 84 studies (46%) that assessed the impact of an intervention, and 66 studies (36%) either described breastfeeding self-efficacy in their cohort, explored associations with demographic variables or used prediction models. Ten studies describing an intervention were classified as descriptive studies, 6 were secondary analyses not involving the intervention and 4 were intervention design / protocol. There were a further 17 studies (9%) that were methodological, assessing the psychometric properties of an assessment tool used in different countries or languages. The final 17 studies (9%) were systematic reviews, meta-analyses, integrative, scoping, or critical reviews (hereinafter referred to as reviews). The studies originated in 34 countries, the majority were high income countries using the World Bank country classification by income level 2023-2024 [53] (Table 1).

**Table 1:** Country income level of included studies (*n* = 184), using World Bank Country classification

High (17 Countries, n = 107, 58%)	Upper Middle (9 Countries, n = 45, 24.5%)	Lower Middle / Low (8 Countries, n = 32, 17.5%)
United States (*n* = 47)	Turkey (*n* = 16)	Lower Middle:
Canada (*n* = 12)	China (*n* = 8)	Iran (*n* = 21)
Australia (*n* = 10)	Indonesia (*n* = 8)	India (*n* = 3)
United Kingdom (*n* = 10)	Brazil (*n* = 5)	Bangladesh (*n* = 2)
Taiwan (*n* = 4)	Malaysia (*n* = 3)	Philippines (*n* = 2)
Hong Kong SAR, China (*n* = 4)	Thailand (*n* = 2)	Cameroon (*n* = 1)
Spain (*n* = 3)	Iraq (*n* = 1)	Egypt (*n* = 1)
Denmark (*n* = 2)	Jordan (*n* = 1)	Myanmar (*n* = 1)
Finland (*n* = 2)	Mexico (*n* = 1)	
Greece (*n* = 2)		Low:
Ireland (*n* = 2)		Ethiopia (*n* = 1)
Netherlands (*n* = 2)		
Portugal (*n* = 2)		
Saudi Arabia (*n* = 2)		
Croatia (*n* = 1)		
Japan (*n* = 1)		
New Zealand (*n* = 1)		

**Fig. 2:**
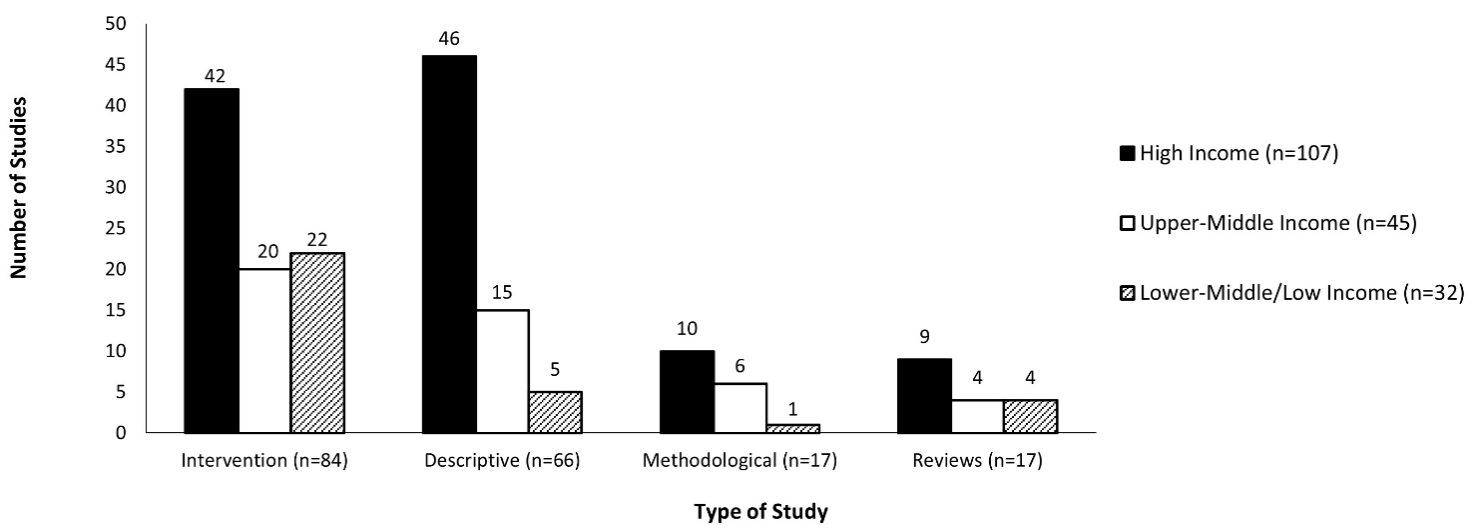
Studies across type and country classification

High income countries conducted most of the studies in each study type category (Fig. 2). The highest proportion of studies conducted in both upper middle and lower middle / low-income countries were intervention studies. There were 22 dissertations included in the review, all of which were conducted in high income countries (16 intervention and 6 descriptive studies).

### Theoretical foundation

Just over half of the studies (*n* = 96, 57%, excluding reviews) described that their choice of measurement tool or design of their intervention was based on a self-efficacy theory or related theories (Table 2). Bandura’s Self-Efficacy Theory [9] / Dennis’ Breastfeeding Self-Efficacy Framework [10] was used most frequently (*n* = 72, 75%), Ajzen’s theories were used 9 times (9.5%), mainly the Theory of Planned Behavior [54], and a variety of other theories were used in 20 studies (21%).

**Table 2:** Theory use across all studies (*n* = 96)

Theory Used & Application (measurement / intervention)	Studies (*n*)
**Bandura: Self Efficacy Theory / Dennis Breastfeeding Self Efficacy Theory**	**72**
Both measurement and intervention	27
Intervention	3
Measurement	37
Mix: Bandura & another theory:	
Bandura & Donabedian (both measurement and intervention)	1
Bandura & Health Action Process Approach (HAPA) (intervention)	1
Bandura & Anderson’s Mutual Caregiving Model (both measurement and intervention)	1
Bandura & Ajzen:	
Measurement	1
Ajzen for intervention & Bandura for measurement	1
**Ajzen**	**9**
Theory of Planned Behavior (TPB), one using extended model (measurement)	6
Sustained Breastfeeding Framework based on Predicting and Changing Behavior Theory (PCBT) (intervention)	1
Mix: Ajzen & Bandura:	
Measurement	1
Ajzen for intervention & Bandura for measurement	1
**Health Promotion Model (HPM)**	**3**
Both measurement and intervention	1
Intervention	2
**Health Belief Model (HBM)**	**2**
Intervention	1
HBM with Integrated Behavioral Model (IBM) (measurement)	1
**Attitude - Social Influence - Self Efficacy Model (ASE): Ajzen & Bandura influences**	**2**
Both measurement and intervention	1
Measurement	1
**Other Theories**	**10**
Breastfeeding Co-Parenting Framework (intervention)	1
IMB: Information-Motivation-Behavior Skill Model (intervention)	1
Mercer’s Theory of Maternal Role Attainment (intervention)	1
Motivation Expectancy Value Theory (both measurement and intervention)	1
Motivation Interviewing Theory (intervention)	1
Self Determination Theory (measurement)	1
Attention, Relevance, Confidence, Satisfaction (ARCS) model of motivational design (intervention)	1
Theory of Community Empowerment (intervention)	1
Watson’s Theory of Human Care (intervention)	1
Behaviour Change Wheel & the Capability, Opportunity and Motivation (COM-B) model of behaviour change (intervention)	1

Of the 84 intervention studies, 44 (52.5%) outlined they were guided by a theory in the design of their intervention. Bandura / Dennis’ Self-Efficacy Theory was the prevalent one in intervention studies (*n* = 33/44, 75%), descriptive studies (*n* = 22/33, 67%), and methodological studies (*n* = 12/13, 92%).

Intervention studies were further examined to explore whether they were more theory-driven in recent years. Although there have been an increasing number of intervention studies conducted in the past decade (*n* = 69 compared with *n* = 15 pre-2013), the proportion of studies incorporating theory has not increased (*n* = 34, 49% compared with *n* = 10, 67% pre-2013) (Supplementary Material Fig. 1).

### Measuring prenatal breastfeeding self-efficacy

There were 164 studies that measured breastfeeding self-efficacy (Table 3) and over 70% used the Breastfeeding Self-Efficacy Scale [42] (*n* = 25), or its short form [43] (*n* = 91). The Breastfeeding Self-Efficacy Scale is designed to measure postpartum breastfeeding self-efficacy and has 33 items with the stem ‘I can always…’ across 3 constructs: breastfeeding technique (e.g. ‘ensure that my baby is properly latched on for the whole feeding’), intrapersonal thoughts (e.g. ‘breastfeed my baby without using formula as a supplement’), and support (e.g. ‘seek out breastfeeding support in my community’). The Breastfeeding Self-Efficacy Scale - Short Form has 14 items from 2 constructs: breastfeeding technique and intrapersonal thoughts. Both scales use a 5-point Likert scale ranging from 1 ‘not at all confident’ to 5 ‘always confident’.

**Table 3:** Tools used to measure breastfeeding self-efficacy (*n* = 164)

Breastfeeding Self-Efficacy measurement tools	No. of Studies
**Breastfeeding Self-Efficacy Scale (BSES) - 33 items**	**25**
Full tool	23
Adapted tool - 5 items used	1
Mix of tools: BSES & BSES-SF	1
**Breastfeeding Self-Efficacy Scale - Short Form (BSES-SF)**	**91**
Full tool	84
Adapted tool - 4 items used	1
Mix of tools: BSES-SF & BSES	1
BSES-SF & PBSES	4
BSES-SF & self-designed	1
**Prenatal Breastfeeding Self-Efficacy Scale (PBSES)**	**18**
Full tool	13
Adapted tool - 5 items used prenatally & 10 items used postpartum	1
Mix of tools: PBSES & BSES-SF	4
**Prenatal Rating of Efficacy in Preparation to Breastfeed (PREP to BF) Scale**	**5**
**Self-designed questionnaire / interview**	**17**
Tool	16
Mix of tools: Self-designed & BSES-SF	1
**Other**:	**8**
Attitude - Social Influence - Self-Efficacy	2
Breastfeeding Attrition Prediction Tool	3
Breastfeeding Personal Efficacy Beliefs Inventory	1
Campbell’s Breastfeeding Scale	1
Maternal Confidence Survey	1
Undefined tool used (3 abstracts, 2 studies - no response from author)	5
No tool used: one question asked to measure self-efficacy	1
Not applicable: 17 reviews & 3 intervention designs	20

Two tools were identified that were specifically designed for use in the prenatal period, Prenatal Breastfeeding Self Efficacy Scale [37] *n* = 18 studies, Prenatal Rating of Efficacy in Preparation to Breastfeed Scale (PREP to BF) [55] *n* = 5 studies, but were much less commonly used (14%). The Prenatal Breastfeeding Self Efficacy Scale has 20 items with the stem ‘I can…’ across 4 themes: skills and demands of breastfeeding (e.g. ‘breastfeed my baby even when I am tired’), gathering information on how to breastfeed and support if needed (e.g. ‘find out what I need to know about breastfeeding my baby’), breastfeeding around other people and feelings of embarrassment (e.g. ‘breastfeed when my family or friends are with me’), and perceived social pressures (e.g. ‘choose to breastfeed my baby even if my family does not want me to’). It uses a 5-point Likert scale ranging from 1 ‘I am definitely not confident’ to 5 ‘I am completely confident’. PREP to BF scale has 39 items with the stem “Thinking about your life right now, how well can you…” across 4 constructs: individual processes (e.g. ‘mentally prepare yourself to breastfeed your baby’), interpersonal processes (e.g. ‘discuss breastfeeding with other mothers or pregnant women’), professional advice (e.g. ‘accept advice from your healthcare provider about breastfeeding’), and social support (e.g. ‘count on your family to support the decisions you make about infant feeding’). All questions are rated on a Likert scale of 0 ‘cannot do at all’ to 10 ‘highly certain can do’.

There were 17 further studies (10%) that used a self-designed questionnaire or interview schedule and 8 studies (5%) that used other measurement tools (Table 3).

The 84 intervention studies mainly used the Breastfeeding Self Efficacy Scales (*n* = 72, 86%) with a small number using prenatal breastfeeding self-efficacy scales (*n* = 9, 10.5%). The 17 methodological studies primarily assessed either the Breastfeeding Self Efficacy Scales (*n* = 9, 53%) or Prenatal Breastfeeding Self Efficacy Scale (*n* = 7, 41%). There was a wider mix of scales used in the 66 descriptive studies, including the highest proportion of ‘other’ scales (*n* = 5, 7.5%) and self-designed measures (*n* = 14, 21%), with Breastfeeding Self Efficacy Scales (*n* = 35, 53%) and prenatal-specific scales (*n* = 9, 13.5%).

**Fig. 3:**
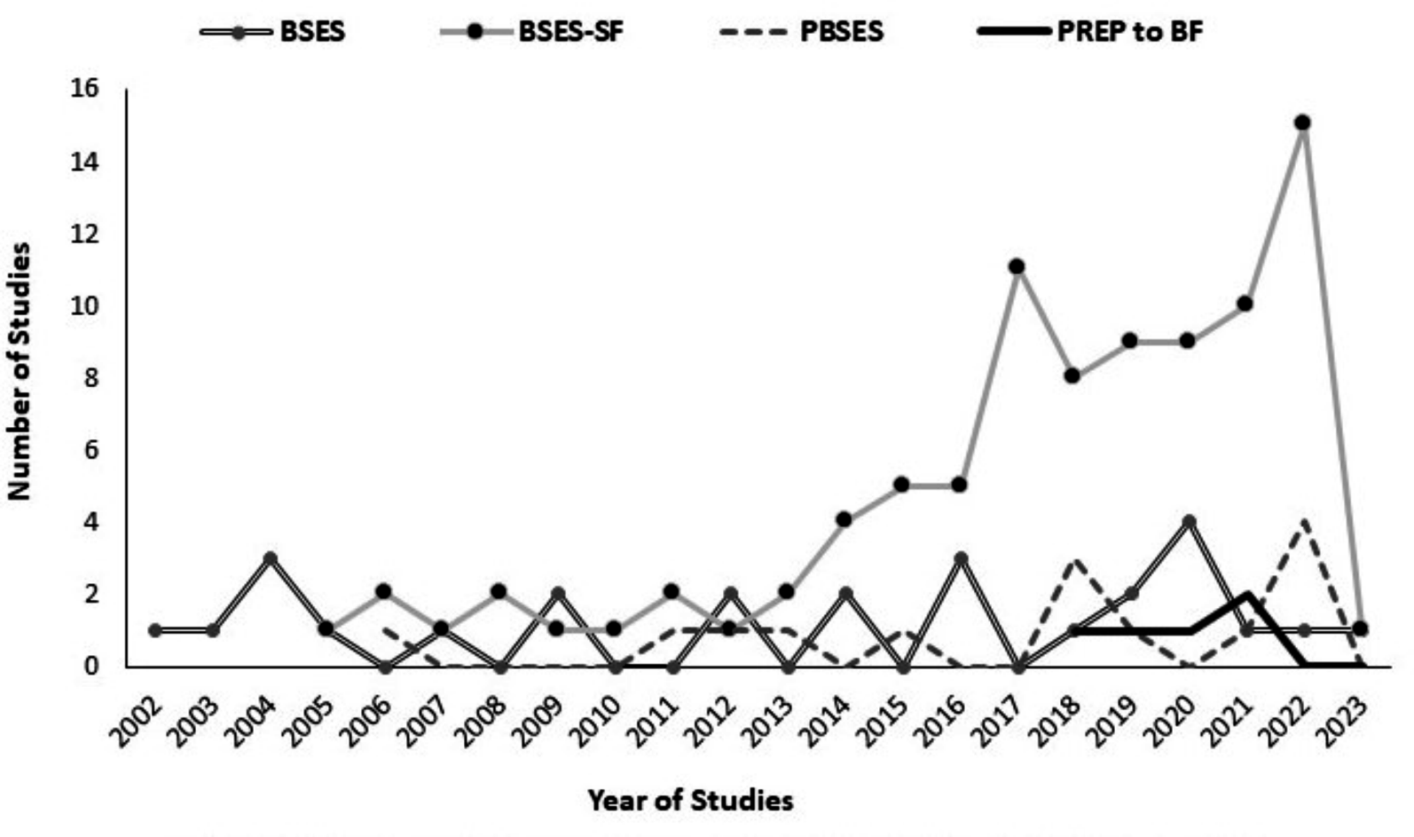
Breastfeeding self-efficacy measurement tools used within studies (2002-2023). Legend: BSES: Breastfeeding Self Efficacy Scale; BSES-SF: Breastfeeding Self Efficacy Scale - Short Form; PBSES: Prenatal Breastfeeding Self Efficacy Scale; PREP to BF: Prenatal Rating of Efficacy in Preparation to Breastfeed Scale

Figure 3 displays the trends in use of the main breastfeeding self-efficacy measurement tools in this review over the past two decades. The original Breastfeeding Self Efficacy Scale has been consistently used since its development in 2002. The short form was used a similar amount for its first 7 years but has grown in popularity since 2013. The Prenatal Breastfeeding Self Efficacy Scale had a slower start with no use in the 4 years following its development in 2006, however its usage is growing, particularly since 2018. The PREP to BF scale is the newest scale and has been used in 5 times within the review timescale (methodological and descriptive studies).

A similar number of studies measured breastfeeding self-efficacy solely in the prenatal period (*n* = 74, 45%) and both prenatal and postpartum periods (*n* = 73, 44.5%). The remaining 10.5% (*n* = 17) were postpartum breastfeeding self-efficacy measurement only. Interventions made up the majority of studies measuring breastfeeding self-efficacy in both periods (*n* = 43, 59%) and all of the postpartum only studies, while prenatal only breastfeeding self-efficacy measurement studies were mostly descriptive studies (*n* = 40, 54%). Most measurement studies (*n* = 96, 65.5%) were in the third trimester, 9.5% (*n* = 14) in the second and 0.5% (*n* = 1) in the first. Nearly a fifth of the studies (*n* = 25, 17%) did not specify the prenatal timepoint and others (*n* = 11, 7.5%) stated that they measured breastfeeding self-efficacy in all trimesters.

The measurement tool used differed depending on the timing of assessment (prenatal, postpartum or both). The studies measuring breastfeeding self-efficacy in both time periods and postpartum only, used the BSES scales (*n* = 63, 86.5% and *n* = 16, 94% respectively). Prenatal period only studies used a wider range of tools including prenatal-specific (*n* = 20, 26.5%), self-designed (*n* = 10, 13.5%) and BSES tools (*n* = 38, 50.5%).

### Other tools

There were 87 studies that used additional assessment tools alongside the breastfeeding self-efficacy measurement, depending on their aims. In total, 145 other tools were used, the most common of which were the Iowa Infant Feeding Attitude Scale (*n* = 16), the Breastfeeding Knowledge Questionnaire (*n* = 9), The Edinburgh Postnatal Depression Scale (*n* = 9), the Breastfeeding Attrition Prediction Tool (*n* = 5), the State-Trait Anxiety Inventory (*n* = 5) and the General Self-Efficacy Scale (*n* = 4). The remainder were used once or twice or were self-designed.

### Interventions

There were 84 intervention studies that aimed to assess its impact on breastfeeding self-efficacy, breastfeeding outcomes, or both. The majority (*n* = 49, 58.5%) occurred solely in the prenatal period, 34 studies (40.5%) spanned the prenatal and postpartum periods, and one was delivered only postpartum. Most of the interventions delivered in the prenatal period commenced in the third trimester (*n* = 59, 71%), with 60.5% of these (*n* = 35) starting towards the middle or end of the trimester. Six interventions (7%) commenced in the second trimester, and the remaining 18 studies (22%) failed to specify the antenatal timepoint.

Over half of the intervention studies (*n* = 44) used a specific theoretical framework. Of those, 23 studies (52%) had high congruence levels (theoretical framework and intervention components described in detail with linkages stated), 12 (27.5%) had medium (theory described but no linkages made with intervention components), and 9 (20.5%) had low (framework named but not described or linked to intervention components).

Intervention design components were grouped into five categories (Supplementary Material Table 2) (1) education (includes written material, lecture/class, smartphone app, interactive computer package, website, activity book, and audio episodes; *n* = 81, 96.5%), (2) encouragement and support (includes counselling, advice, emotional support, reinforcing coping strategies, contact via text messages, Facebook/WhatsApp groups, telephone calls and home visits; *n* = 69, 82%), (3) engagement with participants (includes discussions, questions and answers, problem solving, tailored plan / advice, motivational interviewing, and strategies to overcome issues; *n* = 65, 77.5%) (4) vicarious or kinaesthetic methods of learning (includes video or peer demonstration of breastfeeding, use of dolls and model breasts, role play, and breastfeeding simulation; *n* = 44, 52.5%) and (5) involvement of participant’s social support circle (includes provision of local support group information and encouragement to attend, exploring together how to enhance social support, and involvement of partner or family members in breastfeeding education including how they can support and encourage; *n* = 22, 26%). Theory use and congruence impacted the content of interventions, 77% of high or medium congruence studies (*n* = 27/35) used 4 or 5 components, compared with 34.5% (*n* = 17/49) with low congruence or no theory use.

The component categories were broadly mapped to the four antecedents of self-efficacy theory (Supplementary Material Table 2) for comparison of study outcomes. Most studies incorporated verbal persuasion (through education, encouragement and support, engagement, and involvement of social support circle; *n* = 81, 96.5%); mastery/performance accomplishment (through engagement and vicarious/kinaesthetic learning; *n* = 70, 83.5%); and emotional/physiological states (through engagement and involvement of social support; *n* = 68, 81%). Approximately half of the studies included vicarious experience (through vicarious or kinaesthetic methods of learning; *n* = 44, 52.5%).

### Intervention outcomes

The outcomes reported were either breastfeeding ones such as duration or exclusivity at a particular time point, or breastfeeding constructs, such as self-efficacy, attitudes, knowledge, and intention. Reporting both breastfeeding constructs and outcomes was the most common finding (60.5%, *n* = 51). A further 36% (*n* = 30) and 3.5% of studies (*n* = 3) only reported breastfeeding constructs or breastfeeding outcomes respectively. While statistical analysis is outside the remit of a scoping review, intervention outcomes were categorised according to reported statistical significance. Most interventions reported statistically significant results in breastfeeding self-efficacy (*n* = 58/80, 72.5%) and breastfeeding outcomes (*n* = 38/53, 71.5%). The statistical significance of results varied across the levels of theory applied. Theory-led studies with high or medium congruence (*n* = 35) had 22% more reports of statistically significant results for breastfeeding self-efficacy and 32% more for breastfeeding outcomes. Theory-led studies identified statistically significant results for 85% of breastfeeding self-efficacy (*n* = 29/34) and 86% of breastfeeding outcomes (*n* = 25/29), while those that were not theory-led had significant findings for 63% of breastfeeding self-efficacy (*n* = 29/46) and 54% for breastfeeding outcomes (*n* = 13/24).

More studies reported statistically significant results, particularly breastfeeding outcomes, when the intervention spanned the prenatal and postpartum periods (*n* = 34) compared with prenatal only (*n* = 49). Longer delivery had significant findings for 77.5% of breastfeeding self-efficacy (*n* = 24/31) and 84% of breastfeeding outcomes (*n* = 21/25), while prenatal only were 71% of breastfeeding self-efficacy (*n* = 34/48) and 63% of breastfeeding outcomes (*n* = 17/27). Interventions commencing in the second trimester (*n* = 6) reported statistical significance in 60% of breastfeeding self-efficacy (*n* = 3/5) and 100% (*n* = 4/4) of breastfeeding outcomes, compared with those in the third trimester (*n* = 58) with 82% of breastfeeding self-efficacy results (*n* = 45/55) and 75.5% of breastfeeding outcomes (*n* = 31/41).

The number of intervention components used was also examined. Studies with four or five intervention components reported statistically significant results in 80.5% of breastfeeding self-efficacy (*n* = 33/41) and 82% of breastfeeding outcomes (*n* = 27/33). Studies using three or fewer components reported fewer statistically significant findings for breastfeeding self-efficacy (64%, *n* = 25/39) and breastfeeding outcomes (55%, *n* = 11/20), which were 16.5% and 27% lower than the 4–5 intervention components group. When intervention components were broadly mapped to self-efficacy theory antecedents, those with the potential to address all four antecedents reported more significant results for breastfeeding self-efficacy (*n* = 32/38, 84%) and breastfeeding outcomes (*n* = 25/29, 86%), compared with those with only three (breastfeeding self-efficacy (*n* = 18/28, 64.5%), breastfeeding outcomes (*n* = 11/17, 64.5%)), or two or fewer antecedents (breastfeeding self-efficacy (*n* = 8/14, 57%), breastfeeding outcomes (*n* = 2/7, 28.5%)). When examining interventions with or without a component on participants’ social support, there was no difference in the proportion of reported statistical significance.

### Academy of Breastfeeding Medicine protocols

The Academy of Breastfeeding Medicine clinical protocols [52] are practitioner guidelines to manage common medical problems that may impact breastfeeding success, two of which explicitly refer to prenatal breastfeeding self-efficacy. The ‘Model Maternity Policy Supportive of Breastfeeding’ protocol [56] recommends early (first or second antenatal visit), tailored antenatal breastfeeding support and education, with encouragement for partners and family members to participate. They note that special consideration should be given to behavioural and psychoeducational approaches, and empowerment techniques to increase self-confidence.

The protocol on ‘Breastfeeding Promotion in the Prenatal Setting’ [57] recommends using motivational and self-efficacy support techniques, including exploring knowledge, identifying barriers to reaching goals and other areas where they have successfully reached goals. They suggest strongly considering preconception, prenatal and postnatal components of breastfeeding support and promotion. They state that the first trimester of pregnancy should incorporate the education of partners and support persons about the benefits of breastfeeding and address common barriers including a lack of self-confidence and social support. They also highlight the benefit of social-cognitive theory-based models, competence theory, and workbook-based or group self-efficacy interventions in improving breastfeeding outcomes.

## Discussion

This scoping review provides a synthesis of prenatal breastfeeding self-efficacy literature. We found that only half of studies explicitly used theory to underpin their work and the predominant frameworks used were Bandura’s Self-Efficacy Theory [9] and Dennis’ Breastfeeding Self-Efficacy Framework [10]. Design issues were identified within the included studies in relation to breastfeeding self-efficacy measurement. Most studies used measurement tools designed for postnatal use such as the Breastfeeding Self-Efficacy Scale tools [42, 43], despite prenatal-specific tools being available. Intervention studies showed differences in the timing, content, and theory use within their designs, which impacted the frequency of reported statistical significance for key breastfeeding self-efficacy and breastfeeding outcome results. These findings provide novel insights for consideration in the design and conduct of breastfeeding self-efficacy measurement and interventions.

Prenatal interventions to improve breastfeeding self-efficacy appear to be more effective when a theoretical foundation is used, the breadth of self-efficacy theory is addressed in the intervention design, and delivery spans the prenatal and postpartum periods. Our findings regarding theoretical underpinning align with the results of other systematic reviews conducted on interventions to improve breastfeeding self-efficacy [22, 24, 39, 40, 44, 45]. Bai et al.’s review of theory use in breastfeeding interventions recommends sound application of single or multiple theories to develop effective interventions and evaluate the true impacts on breastfeeding outcomes [44]. Chipojola et al. recommended multiple theories being integrated as they found different theories impacted breastfeeding outcomes in different postpartum periods (Bandura at 1–2 months, Ajzen at 3–6 months) [24]. Skivington et al.’s framework for developing and evaluating complex interventions, recommends that the development or adaptation of interventions should be based on the research evidence and theory of the field [47]. Despite the recommendation to incorporate theory, our review did not find an increase in theory use over time. Therefore, it is important that future studies give greater level of consideration to theoretical underpinning of intervention design.

This review highlights shortfalls in intervention content addressing current literature and Academy of Breastfeeding Medicine recommendations [56, 57] for theory guidance [24, 44, 45] and involvement of partners and family members [58, 59]. Only half of the intervention studies were guided by theory to some degree, a further half of which displayed high congruence of theory with intervention components. Multi-component interventions, particularly those embedding the breadth of self-efficacy theory, appear to be positively associated with statistically significant improvements in breastfeeding self-efficacy and breastfeeding outcomes. However, only half of interventions included components on vicarious or kinaesthetic learning techniques and a quarter provided a focus on the participants’ social support circle. Rollins et al. recognised that family and community are relied on for breastfeeding, as well as professional support and health systems [58]. Bandura noted the interdependence of people and their social structures, recognising that constraints and enabling opportunities can be imposed on the person [60]. The involvement of partners, family members and community provides an opportunity for them to support and enable breastfeeding, but more research is needed on the most effective design and delivery of support in this area. Bartle and Harvey suggest that intention and behaviour may be more influenced by society than personal attitudes and recommend a population-wide approach including partners, family members and healthcare professional in feeding discussions [59]. This may be of particular importance in countries with low breastfeeding rates, where formula feeding is normalised, and societal factors can be a larger barrier to breastfeeding.

Recommendations for early support and promotion [35–38, 56, 57] are not being met by the studies in the review regarding the timescale of breastfeeding self-efficacy measurement and interventions. Most studies, both descriptive and intervention, were conducted in the third trimester of pregnancy. The importance and impact of prenatal timing requires further investigation, particularly as this review shows the six interventions starting in the second trimester reported statistically significant outcomes, and only fifteen studies measured breastfeeding self-efficacy before the third trimester. More evidence is needed to reach meaningful conclusions on the impact of conducting measurements and interventions earlier in pregnancy.

Prenatal breastfeeding self-efficacy measurement requires careful consideration of the theory used and the appropriateness of the tool selected. It is important to incorporate theory into tool design but Bai *et al.’s* review found breastfeeding measurement tools often deviate from the theory guide [44]. Although Breastfeeding Self-Efficacy Scale tools stem from breastfeeding self-efficacy theory [10, 42], the short form focuses on technique and intrapersonal skills, compared with the broader use of theoretical components covered in the prenatally designed Prenatal Breastfeeding Self-Efficacy Scale and PREP to BF tools [37, 55]. The advances in breastfeeding self-efficacy, particularly theory-guided intervention design in the past decade, have likely contributed to the PREP to BF tool design, which although long (39 items), aligns strongly with breastfeeding self-efficacy theory. It is likely that administering a tool that comprehensively addresses breastfeeding self-efficacy theory, even if used independently of an intervention, could raise participant awareness of breastfeeding self-efficacy elements.

The Breastfeeding Self-Efficacy Scale instruments [42, 43], while designed for postpartum use, were predominantly used by the included studies, particularly with interventions. McKinley et al. highlight the importance of phrasing in tool design, recognising the inherent difficulty in seeking a participant’s prenatal confidence levels in breastfeeding techniques they will perform in the future [38], as is the case for some items in the Breastfeeding Self-Efficacy Scales. Bandura’s guide for constructing self-efficacy scales supports the avoidance of estimating confidence in future actions, cautioning that this may lessen the predictive relationship between factors and intention [61]. The predominant use of Breastfeeding Self-Efficacy Scales is likely due to their popularity postpartum, validation in numerous countries and populations, reduced participant demand for the short form version, and direct comparability when using the same tool for prenatal and postpartum assessment. However, Tuthill et al.’s critical review of breastfeeding self-efficacy instruments caution that failure to apply appropriate measures may garner results that are inconclusive, inaccurate, or nonrepresentative of true study effects [62]. They recommend using established tools and adapting them as required [62] and some included studies reported adapting the Breastfeeding Self-Efficacy Scale-Short Form by changing the stem from present tense ‘I can always…’ to future tense ‘I will always…’. Tuthill et al. also recommend refining and improving measurement tools, rather than redefining what already exists [62]. This review indicates the promise of the prenatal tools to assess prenatal breastfeeding self-efficacy, particularly the identification of elements requiring support. More studies are required to establish whether prenatal tools are superior to Breastfeeding Self-Efficacy Scales in assessing prenatal breastfeeding self-efficacy. It is recommended that a tool used across the continuum should incorporate the breadth of self-efficacy theory in its design and use phrasing that allows for applicability to both prenatal and postpartum periods (see Table 4).

**Table 4:** Recommendations and suggestions for future research in prenatal breastfeeding self-efficacy

	Recommendation	Future Research Suggestions
**Measurement**	**Content** • Consider the breadth of self-efficacy theory incorporated into tool design. Wider coverage may provide a more accurate measurement and assist with identifying specific areas requiring prenatal support. • Consider measurement tool content and timing of administration. Postnatal scenario items may be inappropriate for prenatal measurement. • When studies span the prenatal-postpartum continuum, consider phrasing that allows application in both periods. • Intervention studies should consider measurement tools that reflect the intervention content as much as practicable. This may provide more accurate pre- and post-intervention measurement. **Timing** • Consider measuring prenatal breastfeeding self-efficacy early in pregnancy to identify those at-risk of poor breastfeeding outcomes earlier. This will maximise the window of opportunity to provide tailored support in pregnancy.	• Additional studies are needed on theory-driven breastfeeding self-efficacy measurements in early pregnancy. • More extensive use and efficacy testing of tools designed for prenatal use, in research and practice settings. • Investigate the accuracy of existing prenatal breastfeeding self-efficacy tools compared with the more commonly used tools, for identification of areas for tailored support and prediction of initiation and duration. • Assess the impact of early prenatal measurement versus the commonly used late third trimester on breastfeeding initiation and duration.
**Intervention**	**Content** • Consider theoretically underpinning intervention design. • Consider including intervention components involving partners and family members, and methods of vicarious / kinaesthetic learning. • Consider the congruence between the breadth of theory and intervention components used, planning strong connections here in the design phase may lead to improved outcomes. **Timing** • Consider early prenatal intervention to maximise the window of opportunity to provide tailored support and breastfeeding promotion over a longer timeframe. • Consider extending a prenatal intervention into the postnatal period for maximum impact.	• Additional studies are needed on theory-driven interventions to improve breastfeeding self-efficacy in early pregnancy. • Assessment of early versus late prenatal intervention on breastfeeding initiation and duration. • Investigate the most effective design and delivery of social circle support in prenatal intervention. • Investigate intervention components needed prenatally to maximise the impact on breastfeeding outcomes. • Systematic review and meta-analysis of prenatal intervention studies on their incorporation of theory, the type of intervention components employed, and corresponding outcomes.

## Strengths and limitations

The scoping review has a published protocol and followed a robust systematic process. It is not without limitations. These include the categorisation of included studies components and content based on explicit descriptions, which may not align with the original authors categorisation. Some studies that did not focus on breastfeeding self-efficacy were included in the review due to their inclusion of breastfeeding self-efficacy measurement. The quality of included studies varied widely. Some study populations were duplicated because dissertations typically published associated papers with differing analyses on the same data. A broad limitation was the general lack of specification in the title or abstract of the breastfeeding self-efficacy timing which impacted the ability to discern when the measurement or intervention occurred. Preliminary searching gave the impression of a smaller body of work on prenatal breastfeeding self-efficacy, compared with the unexpected number of included studies. Outcomes of intervention studies were summarised according to their reported statistical significance. This is a crude measure lacking sample and effect size, study design or the study quality assessment that a meta-analysis would provide. Intervention component categories were broadly mapped to self-efficacy theory antecedents to support comparison of studies, rather than more comprehensive mapping onto breastfeeding self-efficacy or Behaviour Change Theory frameworks, which was considered beyond the remit of a scoping review.

## Conclusion

This review advances our understanding of prenatal breastfeeding self-efficacy and its central role in improving breastfeeding outcomes. The prenatal period is an important time for breastfeeding self-efficacy measurement, early identification of those at-risk of poor breastfeeding outcomes, and a clear opportunity for providing tailored support and breastfeeding promotion. Prenatal interventions appear to be more effective at improving breastfeeding self-efficacy and breastfeeding outcomes when they incorporate theory-led design, include multiple intervention components, and continue into the postpartum period. Evidence emerging from this review highlights the lack of theoretical underpinning, challenges with breastfeeding self-efficacy measurement tool selection, and measurement and interventions commencing in the third trimester. Further research should aim to incorporate theory into intervention design and use or adapt breastfeeding self-efficacy measurement tools that best meet the study needs. Additional studies are needed on theory-based measurements and interventions delivered in early pregnancy, the uptake and efficacy of existing prenatal breastfeeding self-efficacy tools in practice and research settings, and the intervention components needed to maximise impact on breastfeeding outcomes. Improving breastfeeding self-efficacy through greater attention in early pregnancy has the potential to have an important impact on global breastfeeding rates.

### Electronic supplementary material

Below is the link to the electronic supplementary material.

Supplementary Material 1

Supplementary Material 2

Supplementary Material 3

Supplementary Material 4

## Data Availability

The search string and dataset generated during the current study are available in the Open Science Framework repository, 10.17605/OSF.IO/2JCK7.

## Abbreviations

BSES: Breastfeeding Self-Efficacy Scale
BSES-SF: Breastfeeding Self-Efficacy Scale - Short Form
PBSES: Prenatal Breastfeeding Self-Efficacy Scale
PREP to BF: Prenatal Rating of Efficacy in Preparation to Breastfeed Scale
PRISMA: Preferred Reporting Items for Systematic Reviews and Meta-Analysis
PRISMA-ScR: Preferred Reporting Items for Systematic Reviews and Meta-Analysis extension for Scoping Reviews
WHO: World Health Organization
