# Supplementary material for: An exploration of prenatal breastfeeding self-efficacy: a scoping review
Source: Int J Behav Nutr Phys Act. 2024 Sep 2;21:95. doi: 10.1186/s12966-024-01641-3 (PMC11367871; doi:10.1186/s12966-024-01641-3)
Supplement: Supplementary file 1 — Supplementary Material 1 [file 12966_2024_1641_MOESM1_ESM.docx]

| **Type** | **Author (Year)** | **Country, World Bank Classification** | **Sample, Intervention timing ^a^,  type, #components** | **Tool, timing ^b^** | **Theory (application ^c^), congruence level** | **Outcome type, statistical sig. ^d^** |
| --- | --- | --- | --- | --- | --- | --- |
| Int | Abbass-Dick, J. *et al.* (2017)^1^ | Canada, High | n=66 (31 mothers, 35 fathers), Both*, eHealth resource, 4 | BSES-SF, group of each | Breastfeeding Co-Parenting Framework (I), High | Construct, SE Yes |
| Int | Abuidhail, J. *et al.* (2019)^2^ | Jordan, Upper Middle | n=112, Prenatal, Web-based education, 2 | BSES-SF, Both | Not stated | Construct, SE No |
| Int | Adeboyejo, O.A. (2016)^3^ | US, High | n=54, Prenatal, F2F education, 1 | PBSES, group of each | Bandura/Dennis (Both), Low | Construct, SE No |
| Int | Aghababaei, S. *et al.* (2022)^4^ | Iran, Lower Middle | n=60 (couples), Both, Counselling (GATHER: Greet, Ask, Tell, Help, Explain, and Return), 4 | BSES, Both | Not stated | Construct, SE Yes |
| Int | Amin, S.M. *et al.* (2022)^5^ | Egypt, Lower Middle | n=120 (I:60, C:60), Prenatal, Interactive education, 4 | BSES-SF, Both | Not stated | Construct, SE Yes |
| Int | Ansari, S. *et al.* (2014)^6^ | Iran, Lower Middle | n=130, Prenatal, Group education, 4 | BSES, Both | Bandura (Both), Low | Both, SE Yes, BF Yes |
| Int | Antoñanzas‐Baztán, E. *et al.* (2021)^7^ | Spain, High | n=112 (I:57, C:55), Both, Education (written) & phone, 4 | BSES-SF, Postpartum | Bandura (Both), High | Both, SE No, BF Yes |
| Int | Araban, M. *et al.* (2018)^8^ | Iran, Lower Middle | n=120, Prenatal, Group education & texts, 4 | BSES-SF, Prenatal | Bandura/Dennis (Both), High | Both, SE Yes, BF Yes |
| Int | Azizi, E. *et al.* (2020)^9^ | Iran, Lower Middle | n=46, Prenatal, F2F stress management counselling, 0 | BSES, Both | Bandura (M) | Both, SE Yes, BF Yes |
| Int ^f^ | Bunik, M. *et al.* (2020)^10^ | US, High | n=311 (I:201, C:110), Both, Smartphone app & texts, 3 | Self, Both | Not stated | Construct, SE Yes |
| Int | Campbell, S.H. (1996)^11^ | US, High | n=50 (I:24, C:26), Both, Educational class & phone, 4 | Other, Both | Bandura (I), High | Both, SE Yes, BF Yes |
| Int | Cangöl & Sahin (2017)^12^ | Turkey, Upper Middle | n=67 (I:34, C:33), Both, Motivation program, 3 | BSES-SF, Both | Pender’s Health Promotion Model (I), Medium | Both, SE Yes, BF Yes |
| Int | Chan Man, Y. *et al.* (2016)^13^ | Hong Kong, High | n=71 (I:35, C:36), Both, Interactive workshop, 4 | BSES-SF, Both | Dennis/Bandura (Both), High | Both, SE Yes, BF Yes |
| Int | Chen, S. *et al.* (2022)^14^ | China, Upper Middle | n=148 (I:74, C:74), Both, Couple education (online course) & motivation, 4 | Mix, Both | Info-Motivation-Behavior skill (IMB) model (I), Medium | Both, SE Yes, BF Yes |
| Int | Dal, N.A. *et al.* (2021)^15^ | Turkey, Upper Middle | n=50, Prenatal, Yoga, 0 | BSES-SF, Prenatal | None stated | Construct, SE Yes |
| Int | Damstra, K.M. (2012)^16^ | US, High | n=54, Prenatal, Education, 2 | PBSES, Prenatal | Mix: Bandura & Donabedian (Both), Low | Construct, SE No |
| Int | Dwi Cahyantia, F. *et al.* (2020)^17^ | Indonesia, Upper Middle. | n=38 (I:19, C:19), Prenatal, Group discussions, 3 | BSES-SF, Prenatal | Not stated | Construct, SE Yes |
| Int | Edwards, R.A. *et al.* (2013)^18^ | US, High | n=15 (I:7, C:8), Prenatal, Interactive computer education & motivation, 3 | BSES-SF, Postpartum | Not stated | Construct, SE No |
| Int | El Harit, J. (2015)^19^ | US, High | n=8 (adolescents), Prenatal, Interactive education and small group support, 5 | PBSES, Prenatal | Bandura (Both), High | Construct, SE No |
| Int | Fauzia, F. *et al.* (2020)^20^ | Indonesia, Upper Middle. | n=35, Prenatal, Interactive education, 4 | Not stated, Prenatal | Not stated | Construct, SE Yes |
| Int ^f^ | Fleming, N. *et al.* (2020)^21^ | Canada, High | n=31, Prenatal, Youth informed education, 1 | BSES-SF, Both | Not stated | Both, SE No, BF No |
| Int | Godly-Reynolds, E.M. (2019)^22^ | US, High | n=104 (I:51, C:53), Prenatal, Educational video, 2 | BSES-SF, Both | Bandura (Both), Medium | Construct, SE Yes |
| Int | Grassley, J.S. *et al.* (2017)^23^ | US, High | n=41, Prenatal, Online game-based learning platform, 4 | BSES-SF, Prenatal | Not stated | Construct, SE No |
| Int | Hannula, L.S. *et al.* (2014)^24^ | Finland, High | n=705 (I:431, C:274), Both, Website and support following HCP training, 4 | BSES-SF, Postpartum | Not stated | Both, SE No, BF Yes |
| Int | Harris-Luna & Badr (2018)^25^ | US, High | n=61 (I:31, C:30), Both, Telephone support & resources, 4 | BSES-SF, Prenatal | Theory of Community Empowerment (I), High | Outcome, BF Yes |
| Int ^h^ | Hartati & Hakim (2021)^26^ | Indonesia, Upper Middle | n=36, Prenatal, Educational booklet, 1 | BSES-SF, Prenatal | Not stated | Construct, SE Yes |
| Int | Hatamleh, W. (2012)^27^ | US, High | n=37, Both, Education & phone, 4 | BSES, Both | Bandura (Both), High | Both, SE Yes, BF Yes |
| Int | Hauck, Y. *et al.* (2007)^28^ | Canada, High | n=276 (I:136, C:140), Both, Journal (education & self-completion), 3 | BSES, Postpartum | Bandura (M) | Both, SE No, BF No |
| Int | Heidari, Z. *et al.* (2016)^29^ | Iran, Lower Middle | n=70 (I:35, C:35), Both, Interactive education, counselling & text/calls, 5 | Self, Postpartum | None | Both, SE Yes, BF Yes |

| **Type** | **Author (Year)** | **Country, World Bank Classification** | **Sample, Intervention timing a,  type, #components** | **Tool, timing b** | **Theory (application c), congruence level** | **Outcome type, statistical sig. ^d^** |
| --- | --- | --- | --- | --- | --- | --- |
| Int | Heidary, M. *et al.* (2021)^30^ | Iran, Lower Middle | n=100, Prenatal, Interactive education (couple and spouses’ mother) & support, 5 | BSES, Prenatal | Mix: Belief, Attitude, Subjective Norm, and Enabling Factors (I) & Bandura/Dennis (M), Medium | Construct, SE Yes |
| Int ^f^ | Hmone, M.P. *et al.* (2017)^31^ | Myanmar, Lower Middle | n=353, Both, Promotional texts, 3 | Not stated, Prenatal | Not stated | Outcome, BF Yes |
| Int | Hosseini, S.A. *et al.* (2023)^32^ | Iran, Lower Middle | n=120 (I:60, C:60), Both, Group counselling, 3 | BSES, Prenatal (pre and post) | Bandura (Both), High | Both, SE Yes, BF Yes |
| Int | Iliadou, M. *et al.* (2018)^33^ | Greece, High | n=203, Prenatal, Education, 3 | BSES-SF, Prenatal (pre and post) | Not stated | Construct, SE Yes |
| Int | Ilyas, I. *et al.* (2022)^34^ | Indonesia, Upper Middle. | n=33 (I:17, C:16), Both, Education classes & online counselling, 4 | BSES-SF, Both | Bandura (Both), High | Both, SE No, BF No |
| Int | Ingram, J. (2013)^35^ | UK, High | n=163, Both, Peer support, 3 | BSES-SF, Postpartum | Not stated | Both, SE No, BF N/A qual methods |
| Int | Jackson, N.J. (2014)^36^ | New Zealand, High | n=40, Prenatal, Interactive education, 4 | BSES-SF, Both | Bandura (M) | Both, SE Yes, BF No |
| Int | Javorski, M. *et al.* (2018)^37^ | Brazil, Upper Middle | n=112, Prenatal, Flipchart education, 4 | BSES-SF, Both | Bandura (Both), High | Both, SE Yes, BF Yes |
| Int | Joshi, A. *et al.* (2016)^38^ | US, High | n=46 (I:23, C:23), Postpartum, Computer-based interactive education, 3 | BSES-SF, Both | Sustained Breastfeeding Framework (I), Low | Both, SE No, BF No |
| Int | Kadam, M.P.S. *et al.* (2022)^39^ | India, Lower Middle | n=80, Prenatal, Educational booklet, 2 | Mix, Prenatal | Not stated | Construct, SE No |
| Int | Kamran, A. *et al.* (2012)^40^ | Iran, Lower Middle | n=88 (I:44, C:44), Prenatal, Education, 3 | BSES, Both | Health Belief Model (I), Medium | Both, SE Yes, BF Yes |
| Int | Khorshidifard, M. *et al.* (2017)^41^ | Iran, Lower Middle | n=270 (I F2F:90, I Group:90, C:90), Prenatal, Interactive education, 4 | BSES-SF, Both | Bandura (M) | Both, SE Yes, BF Yes |
| Int | Kluka, S.M. (2004)^42^ | Canada, High | n=209 (I:111, C:98), Prenatal, Interactive workshop & pre-workbook, 4 | BSES, Both | Theory of Maternal Role Attainment (I), Low | Outcome, BF No |
| Int | Kronborg, H. *et al.* (2012)^43^ | Denmark, High | n=1193 (I:603, C:590), Prenatal, 'Ready for Child' programme - education and video, 5 | BSES-SF, Postpartum | Not stated | Both, SE No, BF No |
| Int | Laws, R.A., *et al.* (2023)^44^ | Australia, High | n=266, Both, Hybrid app, education & interaction, 4 | Other, Both | Behaviour Change Wheel & the Capability, Opportunity, and Motivation (COM‐B) model of behaviour change (I), Low | Construct, SE Yes |
| Int | Liu, L. *et al.* (2017)^45^ | China, Upper Middle | n=150 (I:75, C:75), Both, Workshop & counselling, 5 | BSES-SF, Both | Bandura (Both), High | Both, SE Yes, BF Yes |
| Int | Mardiyah, S. *et al.* (2019)^46^ | Indonesia, Upper Middle. | n=80 (I:40, C:40), Prenatal, Peer education, 2 | BSES-SF, Prenatal | Not stated | Construct, SE Yes |
| Int ^f^ | Massa, K. *et al.* (2018)^47^ | US, High | n=335 (I:176, C:159), Prenatal, Activity sheets,1 | Not stated, Both | Not stated | Both, SE No, BF No |
| Int | Meenatchi & Dash (2018)^48^ | India, Lower Middle. | n=90 (I Video:30, I Flip chart:30, C:30), Prenatal, Education, 1 | BSES-SF, Postpartum | Not stated | Construct, SE Yes |
| Int | Mizrak, B. *et al.* (2017)^49^ | Turkey, Upper Middle | n=90 (I:45, C:45), Both, Interactive education & phone counselling, 4 | BSES-SF, Both | Bandura (Both), Medium | Both, SE Yes, BF Yes |
| Int | Mohamad Pilus, F. *et al.* (2022)^50^ | Malaysia, Upper Middle | n=172 (I:86, C:86), Both, Interactive education, support & texts, 4 | BSES-SF, Both | Bandura (Both), High | Construct, SE Yes |
| Int | Mohseni, H. *et al.* (2018)^51^ | Iran, Lower Middle | n=65, Both, Pregnancy care education (BF one part of overall), 1 | BSES, Postpartum | Bandura (M) | Construct, SE Yes |
| Int | Moudi, A. *et al.* (2016)^52^ | Iran, Lower Middle | n=93 (I Peer: 32, I HCP:30, C:31), Both, Education & support, no detail of support provided, 3 | BSES-SF, Both | Bandura (M) | Construct, SE No |
| Int | Naroee, H. *et al.* (2020)^53^ | Iran, Lower Middle | n=140, Prenatal, Motivational interviewing, 3 | BSES-SF, Both | Motivation Interviewing Theory (I), High | Both, SE Yes, BF Yes |
| Int | Nichols, J. *et al.* (2009)^54^ | Australia, High | n=90 (I:45, C:45), Prenatal, Interactive workbook, 4 | BSES, Both | Bandura (Both), High | Both, SE Yes, BF Yes |
| Int | Noel-Weiss J. *et al.* (2006)^55^ | Canada, High | n=110, Prenatal, Interactive workshop, 4 | BSES-SF, Both | Bandura (Both), Medium | Both, SE Yes, BF Yes |
| Int ^f^ | Olenick, P.L. (2006)^56^ | US, High | n=168, Prenatal, Educational class, 1 | BSES-SF, Not stated | Not stated | Both, SE No, BF No |
| Int | Otsuka, K. *et al.* (2014)^57^ | Japan, High | n=781, Prenatal, Educational workbook, 3 | BSES-SF, Both | Bandura (Both), High | Both, SE Yes, BF Yes (in BFHI hospitals) |
| Int | Özturk, C.S. *et al.* (2023)^58^ | Turkey, Upper Middle | n=72, Prenatal, Mandala drawing & BF education, 3 | BSES-SF, Postpartum | Theory of Human Care (I), High | Both, SE Yes, BF No |
| Int | Ozturk, R. *et al.* (2022)^59^ | Turkey, Upper Middle | n=67 (I:34, C:33), Prenatal, Small group education, 4 | BSES-SF, Postpartum | Dennis/Bandura (Both), Low | Both, SE Yes, BF Yes |
| Int | Parmar & Viswanath (2019)^60^ | India, Lower Middle. | n=60 (I:30, C:30), Both, Interactive education & support, 5 | BSES-SF, Both | Dennis/Bandura (Both), High | Both, SE Yes, BF Yes |
| Int | Pate, B.L. (2009)^61^ | US, High | n=23, Both, Peer counsellor emails & support, 3 | BSES-SF, Prenatal | Bandura (M) | Construct, SE Yes |
| Int | Piro & Ahmed (2020)^62^ | Iraq, Upper Middle | n=130 (I:65, C:65), Prenatal, Small group education & booklet, 4 | Mix, Both | Bandura (Both), Medium | Both, SE Yes, BF Yes |
| Int | Poorghasemian, R. *et al.* (2022)^63^ | Iran, Lower Middle | n=100 (I:50, C:50), Both, Counselling (GATHER: Greet, Ask, Tell, Help, Explain & Return), 3 | BSES-SF, Both | Not stated | Both, SE Yes, BF Yes |
| Int | Prasitwattanaseree, P. *et al*. (2019)^64^ | Thailand, Upper Middle | n=83 (I:41, C:42), Both, Interactive education & support, 5 | BSES-SF, Both | Health Promotion Model (I), High | Both, SE Yes, BF Yes |
| Int | Puharić, D. *et al.* (2020)^65^ | Croatia, High | n=400 (I:136, Active Control:128, C:136), Both, Educational booklet & phone, 2 | BSES, Postpartum | Not stated | Both, SE Yes, BF Yes |
| Int | Rabiepoor, S. *et al.* (2019)^66^ | Iran, Lower Middle | n=66 (I:33, C:33), Prenatal, Couple education, training manual & phone counselling, 3 | BSES, Postpartum | Bandura (Both), Low | Construct, SE Yes |
| Int | Reinsma, K. *et al.* (2016)^67^ | Africa - Cameroon, Lower Middle | n=384 (294 women, 90 partners), Prenatal, Education through audio story & discussion (couple), 3 | BSES-SF, Prenatal | Not stated | Construct, SE Yes |
| Int | Reno, R. (2018)^68^ | US, High | n=25, Prenatal, One-to-one motivational activities, 3 | BSES-SF, Prenatal | Not stated | Construct, SE Yes |
| Int | Rippe, M. (2019)^69^ | US, High | n=15, Prenatal, Education & support (HUG: help, understanding, and guidance), 5 | PBSES, Prenatal (pre and post) | Bandura (Both), Medium | Construct, SE No |
| Int | Saljughi, F. *et al.* (2016)^70^ | Iran, Lower Middle | n=74 (I:37, C:37), Prenatal, Role play, 5 | BSES, Both | Not stated | Both, SE Yes, BF Yes |
| Int | Sawyer, R.D. (2022)^71^ | US, High | n=24, Prenatal, Individual-based interactive education, 4 | BSES-SF, Prenatal | Bandura (Both), Medium | Both, SE Yes, BF No |
| Int | Schlickau, J.M. (2005)^72^ | US, High | n=86, Both, Interactive education, 5 | BSES-SF, Both | Health Promotion Model (Both), Medium | Both, SE Yes, BF Yes |
| Int | Scott, J.A. *et al.* (2021)^73^ | Australia, High | n=1,426 fathers (I Class:338, I App:397, I Both class & app:333, C:358), Both*, Fathers educational class, smartphone app or Both, 4 | BSES-SF, Postpartum | Bandura (I), Medium | Both, SE No, BF No |
| Int | Shafaei, F.S. *et al.* (2020)^74^ | Iran, Lower Middle | n=108 (I:54, C:54), Both, Group education & phone, 3 | BSES, Postpartum | Not stated | Both, SE Yes, BF Yes |
| Int ^f^ | Siegel, R. (2004)^75^ | US, High | n=28, Prenatal, Self-hypnosis training (couple), 0 | BSES, Both | Not stated | Both, SE No, BF No |
| Int | Stockdale, J. *et al.* (2008)^76^ | UK - NI, High | n=182 (I:93, C:89), Prenatal, Motivational education class & support, 3 | Self, Postpartum | Motivation / Expectancy Value Theories (Both), High | Both, SE Yes, BF Yes |
| Int | Stokes, L.O. (2019)^77^ | US, High | n=1, Both, Education & phone, 4 | BSES-SF, Prenatal (pre and post) | Bandura (Both), Low | Both, N/A single participant |
| Int ^f^ | Stortini, B. *et al.* (2017)^78^ | Canada, High | n=46 (class:26, peer support:20), Prenatal, Youth-informed education class or peer support, 2 | BSES-SF, Prenatal | Not stated | Construct, SE No |
| Int | Tseng, J-F. *et al.* (2020)^79^ | Taiwan, High | n=93, Prenatal, Interactive couple education and mindfulness training, 5 | BSES-SF, Both | Bandura (Both), High | Both, SE Yes, BF Yes |
| Int | Vincent, A. (2016)^80^ | US, High | n=60 (I:30, C:30), Prenatal, Breastfeeding education class, 3 | BSES-SF, Both | Bandura (M) | Both, SE Yes, BF No |
| Int | Yeh, C-H. (2011)^81^ | Taiwan, High | n=60 (I:30, C:30), Both, Couple education, support & phone, 5 | BSES-SF, Both | Mix: Anderson's Mutual Caregiving Model & Dennis/Bandura (Both), High | Both, SE Yes, BF Yes |
| Int | You, H. *et al.* (2020)^82^ | China, Upper Middle | n=226 women with GDM (I:113, C:113), Individualised education, support & phone, 4 | BSES-SF, Postpartum | Bandura (Both), High | Both, SE Yes, BF Yes |
| Int | Zhao, Y. *et al.* (2020) ^83^ | China, Upper Middle | n=182 (I:91, C:89), F2F couple mental health and BF interactive education & counselling, 5 | BSES, Prenatal | Not stated | Both, SE Yes, BF Yes |
| Int | Zhao, Y. *et al.* (2021)^84^ | China, Upper Middle | n=168 (I:84, C:84), F2F couple mental health and BF interactive education & counselling, 5 | BSES-SF, Both | Not stated | Both, SE Yes, BF Yes |

| **Type** | **Author (Year)** | **Country, World Bank Classification** | **Sample, Intervention timing ^a^,  type, #components** | **Tool, timing ^b^** | **Theory (application ^c^), congruence level** | **Outcome type, statistical sig. ^d^** |
| --- | --- | --- | --- | --- | --- | --- |
| Des | Alyousefi, N. et al. (2022)^85^ | Saudi Arabia, High | n=145 | PBSES, Prenatal | Not stated |  |
| Des | Astuti & Sustiwi (2022)^86^ | Indonesia, Upper Middle | n=91 | BSES-SF, Prenatal | Not stated |  |
| Des | Avery, A. *et al.* (2009)^87^ | US, High | n=152 | Self, Both | Not stated |  |
| Des | Aygör, H. *et al.* (2022)^88^ | Turkey, Upper Middle | n=320 | PBSES, Prenatal | Not stated |  |
| Des | Bailey J. *et al.* (2008)^89^ | UK, High | n=57 | BSES-SF, Both | Bandura |  |
| Des | Bailey, J. (2007)^90^ | UK, High | n=57 | BSES-SF, Both | Bandura |  |
| Des | Bartle & Harvey (2017)^91^ | UK, High | n=149 | BSES-SF, Prenatal | Mix: Ajzen & Bandura |  |
| Des | Başgöl & Küçükkaya (2022)^92^ | Turkey, Upper Middle | n=572 | PBSES, Prenatal | Not stated |  |
| Des | Birk, D.L. (2005)^93^ | US, High | n=61 | BSES, Both | Bandura |  |
| Des | Blyth, R. *et al.* (2002)^94^ | Australia, High | n=300 | BSES, Both | Bandura |  |
| Des | Blyth, R.J. *et al.* (2004)^95^ | Australia, High | n=300 | BSES, Both | Bandura |  |
| Des ^e^ | Brandão, S. *et al.* (2017)^96^ | Portugal, High | Not stated | BSES-SF, Both | Not stated |  |
| Des | Braun, A.L. *et al.* (2019)^97^ | Brazil, Upper Middle | n=470 | BSES-SF, Both | Not stated |  |
| Des | Buxton, K.E. *et al.* (1991)^98^ | US, High | n=187 | Self, Prenatal | Not stated |  |
| Des | Cabieses, B. *et al.* (2014)^99^ | UK, High | n=476 | Self, Prenatal | Ajzen |  |
| Des | Chezem, J. *et al.* (2003)^100^ | US, High | n=74 | Other, Prenatal | Not stated |  |
| Des ^e^ | Chezem & Bolin (2014)^101^ | US, High | n=60 | Self, Prenatal | Not stated |  |
| Des | Chong, A. *et al.* (2016)^102^ | US, High | n=61 | BSES, Both | Role Strain theory |  |
| Des | Corby, K. *et al.* (2021)^103^ | Canada, High | n=401 | BSES-SF, Prenatal | Bandura |  |
| Des | Craig & Dietsch (2010)^104^ | Australia, High | n=10 | Self, Both | Not stated |  |
| Des ^f^ | Davie, P. *et al.* (2022)^105^ | UK, High | n=450 | Self, Prenatal | Not stated |  |
| Des | de Jager, E. *et al.* (2015)^106^ | Australia, High | n=125 | Mix, Both | Not stated |  |
| Des | Demirtaş-Hicyılmaz & Acıkgoz (2017)^107^ | Turkey, Upper Middle | n=187 | BSES-SF, Prenatal | Bandura |  |
| Des | Eddy, T.L. (2015)^108^ | US, High | n=106 | BSES-SF, Both | Not stated |  |
| Des | Efrat, M.W. (2018)^109^ | US, High | n=253 | BSES-SF, Prenatal | Bandura |  |
| Des | Eker & Beşen (2021)^110^ | Turkey, Upper Middle | n=140 | BSES-SF, Prenatal | Not stated |  |
| Des | Erkal Aksoy, Y. *et al.* (2022)^111^ | Turkey, Upper Middle | n=512 | PBSES, Prenatal | Not stated |  |
| Des | Evans N. *et al.* (2021)^112^ | US, High | n=300 | BSES-SF, Prenatal | Health Belief Model (HBM) and Integrated Behavioral Model (IBM) |  |
| Des | Gijsbers, B. *et al.* (2006)^113^ | Netherlands, High | n=89 | Other, Prenatal | Attitude-Social Influence-Self Efficacy (ASE) model |  |
| Des | Gonzales, A.M. (2020)^114^ | Philippines, Lower Middle | n=128 | BSES-SF, Prenatal | Not stated |  |
| Des | Hamid & Zaidi (2020)^115^ | Malaysia, Upper Middle | n=180 | BSES-SF, Prenatal | Bandura |  |
| Des | Herndon, C.H. (2015)^116^ | US, High | n=120 | Other, Prenatal | Ajzen - Theory of Planned Behaviour |  |
| Des | Isik & Cetisli (2022)^117^ | Turkey, Upper Middle | n=104 | BSES-SF, Both | Not stated |  |
| Des | Joshi, A. *et al.* (2015)^118^ | US, High | n=46 | BSES-SF, Both | Not stated |  |
| Des | Khresheh & Ahmed (2018)^119^ | Saudi Arabia, High | n=101 | PBSES, Prenatal | Bandura |  |
| Des | Kools, E.J. *et al.* (2005)^120^ | Netherlands, High | n=373 | Other, Prenatal | Attitude-Social Influence-Self Efficacy (ASE) model |  |
| Des | Laanterä, S. *et al.* (2012)^121^ | Finland, High | n=123 | Self, Prenatal | Not stated |  |
| Des | Lawson & Tulloch (1995)^122^ | Australia, High | n=78 | Self, Prenatal | Not stated |  |
| Des | Lemos Uchôa, J. *et al.* (2014)^123^ | Brazil, Upper Middle | n=50 | BSES-SF, Both | Not stated |  |
| Des | Lodi, J.C. *et al.* (2019)^124^ | Brazil, Upper Middle | n=210 | Mix, Both | Bandura |  |
| Des ^g^ | Lok, K.Y.W. *et al.* (2022)^125^ | Hong Kong, High | n=40 | BSES-SF, Both (protocol) | Not stated |  |
| Des | Martinez-Brockman, J.L. *et al.* (2017)^126^ | US, High | n=119 | Self, Both | Bandura & Health Action Process Approach (HAPA) model |  |
| Des ^e^ | McKinley, Niroula, & Toffoli (2021)^127^ | US, High | n=849 | PREP to BF, Prenatal | Not stated |  |
| Des | McKinley, Knol *et al.* (2021)^128^ | US, High | n=124 | PREP to BF, Prenatal | Bandura |  |
| Des | McKinley, E.M. (2018)^129^ | US, High | n=133 | PREP to BF, Prenatal | Bandura |  |
| Des | Minas & Ganga-Limando (2016)^130^ | South Africa, Low | n=233 | Self, Prenatal | Bandura |  |
| Des | Mitra, A.K. *et al.* (2004)^131^ | US, High | n=656 | Self, Prenatal | Not stated |  |
| Des | Mossman, M. *et al.* (2008)^132^ | Canada, High | n=100 | BSES-SF, Both | Bandura |  |
| Des | Nilsson, I.M.S. *et al.* (2020)^133^ | Denmark, High | n=2,804 | None, Both | Bandura |  |
| Des | Nisa’, F. et al. (2021)^134^ | Indonesia, Upper Middle | n=108 | Not stated, Prenatal | Not stated |  |
| Des | Noel-Weiss, J. *et al.* (2006)^135^ | Canada, High | n=16 | N/A - design of intervention | Bandura |  |
| Des | O’Sullivan, E.J. *et al.* (2019)^136^ | Ireland, High | n=100 | BSES-SF, Both | Not stated |  |
| Des | O'Campo, P. *et al.* (1992)^137^ | US, High | n=198 | Self, Both | Not stated |  |
| Des | Özdemir, F. *et al.* (2022)^138^ | Turkey, Upper Middle | n=54 | BSES-SF, Prenatal | Not stated |  |
| Des | Risica & McCausland (2017)^139^ | US, High | n=399 | Self, Prenatal | Not stated |  |
| Des | Robinson & Vandevusse (2011)^140^ | US, High | n=59 | PBSES, Prenatal | Bandura |  |
| Des | Serrano-Alvarado, K. *et al.* (2022)^141^ | Mexico, Upper Middle | n=728 | BSES-SF, Prenatal | Ajzen - Theory of Planned Behaviour |  |
| Des | Shayle, A. (2015)^142^ | UK, High | n=63 | BSES-SF, Prenatal | Bandura |  |
| Des | Shipp, G.M. *et al.* (2022)^143^ | US, High | n=53 | BSES-SF, Both | Not stated |  |
| Des | Stockdale, J. *et al.* (2014)^144^ | UK, High | N/A | N/A - design of intervention | Attention, Relevance, Confidence, Satisfaction (ARCS) model of motivational design |  |
| Des | Thomas, J. *et al.* (2015)^145^ | Bangladesh, Lower Middle | n=2,400 | Self, Prenatal | Ajzen - Theory of Planned Behaviour |  |
| Des ^e^ | Wilhelm & Aguirre (2015)^146^ | US, High | n=12 | BSES-SF, Prenatal | Not stated |  |
| Des | Wu, S.-F.V. *et al.* (2021)^147^ | Taiwan, High | n=120 | BSES-SF, Prenatal | Not stated |  |
| Des | Yazdanpanah, F. *et al.* (2022)^148^ | Iran, Lower Middle | n=249 | BSES-SF, Prenatal | Theory of Planned Behaviour & Extended model |  |
| Des | Yu, E.A. *et al.* (2015)^149^ | Bangladesh, Lower Middle | n=2,400 | Other, Both | Ajzen - Theory of Planned Behaviour |  |
| Des | Zhu, J. *et al.* (2014)^150^ | China, Upper Middle | n=201 | BSES, Prenatal | Bandura |  |
| Meth | Alus Tokat M. *et al.* (2010)^151^ | Turkey, Upper Middle | n=294 | BSES-SF, group of each | Bandura |  |
| Meth | Aydin & Pasinlioglu (2018)^152^ | Turkey, Upper Middle | n=326 | PBSES, Prenatal | Not stated |  |
| Meth | Brandão, S. *et al.* (2018)^153^ | Portugal, High | n=373 | BSES-SF, Prenatal | Bandura |  |
| Meth | Cleveland & McCrone (2005)^154^ | US, High | n=479 | BPEBI, Prenatal | Bandura |  |
| Meth | Creedy, D.K. *et al.* (2003)^155^ | Australia, High | n=300 | BSES, Both | Bandura |  |
| Meth | Dennis, C.-L. *et al.* (2011)^156^ | Canada, High | n=103 | BSES-SF, Both | Bandura |  |
| Meth | Evcili & Demirel (2020)^157^ | Turkey, Upper Middle | n=407 | PREP to BF, Prenatal | Bandura |  |
| Meth | Hazar & Akça (2018)^158^ | Turkey, Upper Middle | n=200 | PBSES, Prenatal | Not stated |  |
| Meth | Husin, H. *et al.* (2017)^159^ | Malaysia, Upper Middle | n=205 | BSES-SF, group of each | Bandura |  |
| Meth | Iliadou, M. *et al.* (2020)^160^ | Greece, High | n=173 | BSES-SF, Both | Bandura |  |
| Meth | Lau, C.Y.K. *et al.* (2017)^161^ | Hong Kong, High | n=591 | N/A - development of tool | Self Determination Theory |  |
| Meth | McKinley, E.M. *et al.* (2019)^162^ | US, High | n=124 | PREP to BF, Prenatal | Bandura |  |
| Meth | Oriá M.O.B. *et al.* (2009)^163^ | Brazil, Upper Middle | n=117 | BSES, Prenatal | Bandura |  |
| Meth | Piñeiro-Albero, R.M. *et al.* (2013)^164^ | Spain, High | n=234 | PBSES, Prenatal | Not stated |  |
| Meth | Shahry, P. *et al.* (2021)^165^ | Iran, Lower Middle | n=510 | BSES-SF, Both | Bandura |  |
| Meth | Silva‐Tubio, J.R. *et al.* (2021)^166^ | Spain, High | n=1,218 | PBSES, Prenatal | Not stated |  |
| Meth | Wells K.J. *et al.* (2006)^167^ | US, High | n=279 | PBSES, Prenatal | Bandura |  |

**Continued…**

**Supplementary Table 1: (continued)**

| **Type** | **Author (Year)** | **Country, World Bank Classification** | **Sample** | **Findings** |
| --- | --- | --- | --- | --- |
| Rev | Bai, Y.K. *et al.* (2019)^168^ | US, High | n=18 studies | Theory use in breastfeeding interventions - most interventions on BSE, self-efficacy studies used theory in measurement, others inconsistent. Relationships between constructs and outcomes mostly not tested. |
| Rev | Brockway, M. *et al.* (2017)^169^ | Canada, High | n=11 studies | SR & MA: Success of BSE interventions and impact on rates - effective increasing rates @ 1 and 2 months pp, most success using education and multiple settings and contact points, researchers should consider interventions that use BSE as a social change theory. |
| Rev | Chambers, J.A. *et al.* (2007)^170^ | UK, High | n=22 studies | SR: Measures of BSE, attitudes, knowledge, and satisfaction reveal that none are ideal for UK population. Trend is to create new rather than evaluate and improve existing. |
| Rev | Chipojola, R. *et al.* (2020)^171^ | Taiwan, High | n=24 studies | SR & MA: Impact of theory-based educational interventions on BF outcomes - increased BSE in early postpartum and exclusive BF for up to 6 months. |
| Rev | Cummins, L. *et al.* (2022)^172^ | Australia, High | n=26 studies | Integrative review of factors influencing exclusive BF among women with GDM - 4 categories: personal, antenatal, intrapartum and postnatal factors. BF intention, confidence and support no different to general population. |
| Rev | Galipeau, R. *et al.* (2018)^173^ | Canada, High | n=17 studies | SR & MA: Effect of interventions on BSE and received insufficient milk supply - significant effect on BF cessation under 6 months (but not 4-6 weeks), and exclusive BF cessation at both 4-6 weeks and under 6 months. |
| Rev | Ghasemi, V. *et al.* (2019)^174^ | Iran, Lower Middle | n=21 studies | SR: Effect of intervention on BSE using Bandura theory - significantly higher BSE in intervention group. |
| Rev | Kehinde, J. *et al.* (2023)^175^ | Ireland, High | n=14 studies | SE: Effect of prenatal BF education on BF - general correlation found, high positive BF outcomes in studies attributed to prenatal BF education. |
| Rev | Maleki, A. *et al.* (2021)^176^ | Iran, Lower Middle | n=40 studies | SR & MA: Effect of educational intervention on BSE - recommended that interventions are better based on the theory, in healthcare setting, a group class format, during pregnancy, with direct method format, and continued to the first week of postpartum. |
| Rev | Morado Gonzales Jr, A. (2021)^177^ | Philippines, Lower Middle | n=34 studies | Scoping review of BSE in Asia and Pacific - education has positive effect on BSE and exclusive BF duration. Health care institutions could promote interventions regarding positive breastfeeding experiences. |
| Rev | Prasopkittikun & Sangperm (2017)^178^ | Thailand, Upper Middle | n=10 studies | Integrative review of BSE interventions for BF outcomes in Thailand - indicated the positive effects of self-efficacy promoting interventions on the breastfeeding outcomes |
| Rev | Qian, J. *et al.* (2021)^179^ | China, Upper Middle | n=15 studies | SR & MA: Effect of mHealth-based interventions on BF status (RCTs) - significant increase in exclusive BF rate, BSE, BF attitude, and reduced health problems in infant. |
| Rev | Rahmadani & Rahmawati (2022)^180^ | Indonesia, Upper Middle | n=15 studies | MA: Effect of BF education on BSE and exclusive BF - education programs for pregnant women affect BSE and exclusive BF. |

| **Type** | **Author (Year)** | **Country, World Bank Classification** | **Sample** | **Findings** |
| --- | --- | --- | --- | --- |
| Rev | Seddighi, A. *et al.* (2022)^181^ | Iran, Lower Middle | n=25 studies | SR: Educational interventions to improve BSE - several studies suggest prenatal support through various educational methods is essential predictor of BSE. Modalities were grouped into visual aids, verbal education, and telephone support. Demonstrates importance of designing interventions to improve BSE. |
| Rev | Wong, M.S. *et al.* (2021)^182^ | Hong Kong, High | n=13 studies | SR & MA: Effect of educational and supportive interventions on BF and BSE - benefit on exclusive BF at least 6 months, and partial BF and BSE at 2 months. Suggests optimal approach is continuous pre to postnatal support with teaching and telephone follow-ups. |
| Rev | Wood, N.K. *et al.* (2016)^183^ | US, High | n=6 studies | SR: Interventions that enhance BF initiation, duration and exclusivity - knowledge and skills, emotional support and BSE addressed in studies, but mothers had difficulty transferring to their BF practice. |
| Rev | Wu, W. *et al.* (2021)^184^ | China, Upper Middle | n=22 studies | Qualitative meta-aggregation: Factors influencing BF practices in China - potential benefit of family members influence on BSE, maximise role of primary care in promotion, standardise training to promote evidenced based approaches, increase facilities for pumping. |

**References**

1. Abbass-Dick J, Xie F, Koroluk J, Alcock Brillinger S, Huizinga J, Newport A, et al. The Development and piloting of an eHealth breastfeeding resource targeting fathers and partners as co-parents. Midwifery. 2017;50:139-47.

2. Abuidhail J, Mrayan L, Jaradat D. Evaluating effects of prenatal web-based breastfeeding education for pregnant mothers in their third trimester of pregnancy: Prospective randomized control trial. Midwifery. 2019;69:143-9.

3. Adeboyejo OA. Breastfeeding Education for Women with Diabetes, Pregnancy-Induced Hypertension, and Multiple Gestations. Breastfeeding Education for Women with Diabetes, Pregnancy-induced Hypertension & Multiple Gestations. 2016:1-.

4. Aghababaei S, Khodakarami B, Farhadian M, Kamali M. Promoting maternal self efficacy and paternal attitudes towards breastfeeding: a randomized controlled trial. GLOBAL HEALTH PROMOTION. 2022:17579759221091194.

5. Amin SM, Mahrous ES, Alrimawi I, Elbialy AA. The effectiveness of an interactive digital-based educational program in improving breastfeeding knowledge, attitudes and self-efficacy among primiparous women in Egypt. African Journal of Reproductive Health. 2022;26(11):79-91.

6. Ansari S, Abedi P, Hasanpoor S, Bani S. The Effect of Interventional Program on Breastfeeding Self-Efficacy and Duration of Exclusive Breastfeeding in Pregnant Women in Ahvaz, Iran. int. 2014;2014:510793.

7. Antoñanzas-Baztán E, Belintxon M, Marín-Fernández B, Redín-Areta MD, Mujika A, Pumar-Méndez MJ, et al. Six-month breastfeeding maintenance after a self-efficacy promoting programme: an exploratory trial. Scand J Caring Sci. 2021;35(2):548-58.

8. Araban M, Karimian Z, Kakolaki ZK, McQueen KA, Dennis CL. Randomized controlled trial of a prenatal breastfeeding self-efficacy intervention in primiparous women in Iran. Journal of Obstetric, Gynecologic, &amp; Neonatal Nursing. 2018;47(2):173-83.

9. Azizi E, Maleki A, Mazloomzadeh S, Pirzeh R. Effect of Stress Management Counseling on Self-Efficacy and Continuity of Exclusive Breastfeeding. Breastfeed Med. 2020;15(8):501-8.

10. Bunik M, Jimenez-Zambrano A, Beaty B, Zhang X, Moore S, Bull S, et al. Mother's milk messagingb (Mmm): Mixed methods evaluation of bilingual app and texting program to support breastfeeding (BF). Breastfeeding Medicine. 2020;15(10):A25-A6.

11. Campbell SH. Breastfeeding self-efficacy: The effects of a breastfeeding promotion nursing intervention [Ph.D.]. Ann Arbor: University of Rhode Island; 1996.

12. Cangöl E, Şahin NH. The Effect of a Breastfeeding Motivation Program Maintained During Pregnancy on Supporting Breastfeeding: A Randomized Controlled Trial. Breastfeed Med. 2017;12:218-26.

13. Chan Man Y, Ip Wan Y, Choi Kai C. The effect of a self-efficacy-based educational programme on maternal breast feeding self-efficacy, breast feeding duration and exclusive breast feeding rates: A longitudinal study. Midwifery. 2016;36:92-8.

14. Chen S, Li L, Sun Q, Chen S, Cheng J, Xiong S. Effect of IMB Model Combined with Spousal Support Breastfeeding Intervention on PBSES Score and Breastfeeding Rate of Primipara with Chronic Hepatitis B Virus Infection. BioMed Research International. 2022;2022:1-8.

15. Dal NA, Gümüşsoy S, Sarıdoğan E, Özdemir Ç. The effect of pregnancy yoga on breastfeeding self-efficacy and prenatal attachment: A semi-experimental study. International Medicine. 2021;3(3):80-5.

16. Damstra KM. Improving breastfeeding knowledge, self-efficacy and intent through a prenatal education program. 2012.

17. Dwi Cahyantia F, Nimah L, Pradaniea R. The effect of buzz group modification method on exclusive breastfeeding against self efficacy in pregnant women: A quasi experiment. Medico-Legal Update. 2020;20(3):446-51.

18. Edwards RA, Bickmore T, Jenkins L, Foley M, Manjourides J. Use of an interactive computer agent to support breastfeeding 2013.

19. El Harit J. The effect of an antenatal breastfeeding intervention on breastfeeding self-efficacy and intention among Inner City adolescents. 2015.

20. Fauzia F, Ekayanthi NWD, Fudholi A. The influence of participative counseling approach on improving breastfeeding self-efficacy to pregnant women in Indonesia. Asian Journal of Pharmaceutical and Clinical Research. 2020;13(1):85-8.

21. Fleming N, Cantin C, Peterson W. Improving breastfeeding outcomes using an innovative youth-informed breastfeeding program for young women. Journal of Obstetrics and Gynaecology Canada. 2020;42(5):674.

22. Godly-Reynolds EM. Evaluating a Prenatal, Breastfeeding Educational Video’s Influence on Protective Resources, Psychosocial Factors, and Breastfeeding Outcomes [M.A.]. Ann Arbor: The University of North Carolina at Charlotte; 2019.

23. Grassley JS, Connor KC, Bond L. Game-based online antenatal breastfeeding education: A pilot. Appl Nurs Res. 2017;33:93-5.

24. Hannula LS, Kaunonen ME, Puukka PJ. A study to promote breast feeding in the Helsinki Metropolitan area in Finland 2014.

25. Harris-Luna ML, Badr LK. Pragmatic Trial to Evaluate the Effect of a Promotora Telephone Intervention on the Duration of Breastfeeding. J Obstet Gynecol Neonatal Nurs. 2018;47(6):738-48.

26. Hartati S, Hakim N. A New Exclusive Breastfeeding Booklet to Improve Self-Efficacy. KnE Life Sciences. 2021:870-80.

27. Hatamleh W. Prenatal breastfeeding intervention program to increase breastfeeding duration among low income women. Health. 2012;4(3):143-9.

28. Hauck Y, Hall WA, Jones C. Prevalence, self-efficacy and perceptions of conflicting advice and self-management: effects of a breastfeeding journal. J Adv Nurs. 2007;57(3):306-17.

29. Heidari Z, Keshvari M, Kohan S. Clinical trial to comparison the effect of family-centered educational-supportive program on mothers' empowerment in breast-feeding. International Journal of Pediatrics. 2016;4(3):1445-51.

30. Heidary M, Akbarzadeh M, Ahmadinezhad F. Impacts of Antenatal Educational Interventions base on BASNEF Model on Mothers' Breastfeeding Self-Efficacy: A Quasi-Experimental Study. International Journal of Multicultural and Multireligious Understanding. 2021;8(7):28-36.

31. Hmone MP, Li M, Agho K, Dibley M. Impact of SMS text messages to improve exclusive breastfeeding and reduce other adverse infant feeding practices in yangon, Myanmar: A randomized controlled trial. Annals of Nutrition and Metabolism. 2017;71:610-1.

32. Hosseini SA, Vakilian K, Shabestari AA, Nokani M, Almasi A. Effect of Midwife-led Breastfeeding Counseling based on Bandura's Model on Self-efficacy and Breastfeeding Performance: An Educational Trial Study. The Open Public Health Journal. 2023;16(1).

33. Iliadou M, Lykeridou K, Prezerakos P, Swift EM, Tziaferi SG. Measuring the effectiveness of a midwife led education programme in terms of breastfeeding knowledge and self-efficacy, attitudes towards breastfeeding, and perceived barriers of breastfeeding among pregnant women. Materia Socio Medica. 2018;30(4):240-5.

34. Ilyas I, Citrakesumasari, Hadju V, Salam A, Abdullah HMT, Aspar M. Breastfeeding Education Package for Working Women to Increase Breastfeeding Self-Efficacy, and Breastfeeding Outcome in Tanjungpinang City. INTERNATIONAL JOURNAL OF EARLY CHILDHOOD SPECIAL EDUCATION. 2022;14(3):345-56.

35. Ingram J. A mixed methods evaluation of peer support in Bristol, UK: mothers', midwives' and peer supporters' views and the effects on breastfeeding. BMC Pregnancy Childbirth. 2013;13:192.

36. Jackson NJ. The Impact of Antenatal Breastfeeding Education on Young Women’s Breastfeeding Self-efficacy and Breastfeeding Rates 2014.

37. Javorski M, Rodrigues AJ, Dodt RCM, Almeida PC, Leal LP, Ximenes LB. Effects of an educational technology on self-efficacy for breastfeeding and practice of exclusive breastfeeding. Rev Esc Enferm USP. 2018;52:e03329.

38. Joshi A, Amadi C, Meza J, Aguire T, Wilhelm S. Evaluation of a computer-based bilingual breastfeeding educational program on breastfeeding knowledge, self-efficacy and intent to breastfeed among rural Hispanic women. Int J Med Inform. 2016;91:10-9.

39. Kadam MPS, Patil N, Salunkhe J. A Study To Assess The Impact Of Nursing Intervention On Mother’s Breastfeeding Self-Efficacy Among Primiparous Mothers At Tertiary Care Hospital. Journal of Pharmaceutical Negative Results. 2022:6150-8.

40. Kamran A, Shrifirad G, Mirkarimi SK, Farahani A. Effectiveness of breastfeeding education on the weight of child and self-efficacy of mothers - 2011. J Educ Health Promot. 2012;1:11.

41. Khorshidifard M, Amini M, Dehghani MR, Zaree N, Pishva N, Zarifsanaiey N. Assessment of Breastfeeding Education by Face to Face and Small-Group Education Methods in Mothers’ Self-Efficacy in Kazeroun Health Centers in 2015. Women’s Health Bulletin. 2017;4(3):1-6.

42. Kluka SM. A randomized controlled trial to test the effect of an antenatal educational intervention on breastfeeding duration among primiparous women [Ph.D.]. Ann Arbor: The University of British Columbia (Canada); 2004.

43. Kronborg H, Maimburg RD, Vaeth M. Antenatal training to improve breast feeding: a randomised trial. Midwifery. 2012;28(6):784-90.

44. Laws RA, Cheng H, Rossiter C, Kuswara K, Markides BR, Size D, et al. Perinatal support for breastfeeding using mHealth: A mixed methods feasibility study of the My Baby Now app. Maternal & Child Nutrition. 2023;19(2):e13482.

45. Liu L, Zhu J, Yang J, Wu M, Ye B. The Effect of a Perinatal Breastfeeding Support Program on Breastfeeding Outcomes in Primiparous Mothers. West J Nurs Res. 2017;39(7):906-23.

46. Mardiyah S, Anggorowati, Nurrahima A. Effects of peer education on improving self-efficacy of pregnant women in breastfeeding the baby. Pakistan Journal of Medical and Health Sciences. 2019;13(4):1282-5.

47. Massa K, Amorado P, Miller C, Gavard JA, Shyken J. Do self-help-style activity sheets promote breastfeeding among pregnant mothers? Obstetrics and Gynecology. 2018;131:92S.

48. Meenatchi B, Dash MB. Effectiveness of Prenatal Breastfeeding Self-efficacy Intervention Program on Successful Breastfeeding among the Primi mothers Admitted to RGGW and CH, Puducherry. 2018.

49. Mizrak B, Ozerdogan N, Colak E. The Effect of Antenatal Education on Breastfeeding Self-Efficacy: Primiparous Women in Turkey. International Journal of Caring Sciences. 2017;10(1):503-10.

50. Mohamad Pilus F, Ahmad N, Mohd Zulkefli NA, Mohd Shukri NH. Effect of Face-to-Face and WhatsApp Communication of a Theory-Based Health Education Intervention on Breastfeeding Self-Efficacy (SeBF Intervention): Cluster Randomized Controlled Field Trial. JMIR Mhealth Uhealth. 2022;10(9):e31996.

51. Mohseni H, Jahanbin I, Sekhavati E, Tabrizi R, Kaviani M, Ghodsbin F. An investigation into the effects of prenatal care instruction at home on breast-feeding self-efficacy of first-time pregnant women referred to shiraz clinics, Iran. International Journal of Women's Health and Reproduction Sciences. 2018;6(1):41-6.

52. Moudi A, Tafazoli M, Boskabadi H, Ebrahimzadeh S, Salehiniya H. Comparing the Effect of Peer Support and Training by Healthcare Providers on Women’s Breastfeeding Self-Efficacy. Journal of Midwifery and Reproductive Health. 2016;4(1):488-97.

53. Naroee H, Rakhshkhorshid M, Shakiba M, Navidian A. The effect of motivational interviewing on self-efficacy and continuation of exclusive breastfeeding rates: a quasi-experimental study. Breastfeeding Medicine. 2020;15(8):522-7.

54. Nichols J, Schutte NS, Brown RF, Dennis CL, Price I. The impact of a self-efficacy intervention on short-term breast-feeding outcomes. Health Educ Behav. 2009;36(2):250-8.

55. Noel-Weiss J, Rupp A, Cragg B, Bassett V, Woodend AK. Randomized controlled trial to determine effects of prenatal breastfeeding workshop on maternal breastfeeding self-efficacy and breastfeeding duration. J Obstet Gynecol Neonatal Nurs. 2006;35(5):616-24.

56. Olenick PL. The effect of structured group prenatal education on breastfeeding confidence, duration and exclusivity to twelve weeks postpartum: Touro University International; 2006.

57. Otsuka K, Taguri M, Dennis C-L, Wakutani K, Awano M, Yamaguchi T, et al. Effectiveness of a breastfeeding self-efficacy intervention: Do hospital practices make a difference? Maternal and Child Health Journal. 2014;18(1):296-306.

58. Ozturk CS, Demir K. The Effect of Mandala Activity and Technology-Based Breastfeeding Program on Breastfeeding Self-Efficacy and Mother-Infant Attachment of Primiparous Women: A Randomized Controlled Study. J Med Syst. 2023;47(1):44.

59. Ozturk R, Ergun S, Ozyazicioglu N. Effect of antenatal educational intervention on maternal breastfeeding self-efficacy and breastfeeding success: a quasi-experimental study. Revista Da Escola de Enfermagem Da Usp. 2022;56:e20210428.

60. Parmar S, Viswanath L. The Effectiveness of a Breastfeeding Self-Efficacy Programme on Breastfeeding Self-Efficacy and Breastfeeding Practice among Primigravida Mothers. International Journal of Nursing Care. 2019;7(1):69-73.

61. Pate BL. Effectiveness of Web-based programs in improving breastfeeding self-efficacy: University of Arkansas for Medical Sciences; 2009.

62. Piro SS, Ahmed HM. Impacts of antenatal nursing interventions on mothers' breastfeeding self-efficacy: an experimental study. BMC Pregnancy Childbirth. 2020;20(1):19.

63. Poorghasemian R, Vakilian K, Khorsandi M. The Effect of Counseling on Breastfeeding Self-Efficacy (BSFE) in Women with Gestational Diabetes. Current Women's Health Reviews. 2022;18(3):94-101.

64. Prasitwattanaseree P, Sinsuksai N, Prasopkittikun T, Viwatwongkasem C. Effectiveness of Breastfeeding Skills Training and Support Program among First Time Mothers: A Randomized Control Trial. Pacific Rim International Journal of Nursing Research. 2019;23(3):258-70.

65. Puharić D, Malički M, Borovac JA, Šparac V, Poljak B, Aračić N, et al. The effect of a combined intervention on exclusive breastfeeding in primiparas: A randomised controlled trial. Matern Child Nutr. 2020;16(3):e12948.

66. Rabiepoor S, Khodaei A, Valizadeh R. Husbands' participation in prenatal care and breastfeeding self-efficacy in Iranian women: A randomized clinical trial. Med J Islam Repub Iran. 2019;33:58.

67. Reinsma K, Bolima N, Fonteh F, Okwen P, Siapco G, Yota D, et al. Bobbi Be Best: the development and evaluation of an audio program and discussion guide to promote exclusive breastfeeding in Cameroon, Central Africa. Global Health Promotion. 2016;23(3):14-26.

68. Reno R. A Pilot Study of a Culturally Grounded Breastfeeding Intervention for Pregnant, Low-Income African American Women. J Hum Lact. 2018;34(3):478-84.

69. Rippe M. Addressing Prenatal Breastfeeding Self-efficacy and Advancing the Baby Friendly Hospital Initiative with HUG Your Baby Curriculum 2019.

70. Saljughi F, Esfahani MS, Kohan S, Ehsanpour S. Promoting breastfeeding self-efficacy through role-playing in pregnant women. International Journal of Pediatrics. 2016;4(7):2061-8.

71. Sawyer RD. Improving maternal breastfeeding self-efficacy and exclusive breast milk feeding outcomes in first-time mothers using prenatal breastfeeding self-efficacy education: ProQuest Information & Learning; 2022.

72. Schlickau JM. Prenatal breastfeeding education: An intervention for pregnant immigrant Hispanic women [Ph.D.]. United States -- Nebraska: University of Nebraska Medical Center; 2005.

73. Scott JA, Burns SK, Hauck YL, Giglia RC, Jorgensen AM, White BK, et al. Impact of a Face-To-Face Versus Smartphone App Versus Combined Breastfeeding Intervention Targeting Fathers: Randomized Controlled Trial. JMIR Pediatr Parent. 2021;4(2):e24579.

74. Shafaei FS, Mirghafourvand M, Havizari S. The effect of prenatal counseling on breastfeeding self-efficacy and frequency of breastfeeding problems in mothers with previous unsuccessful breastfeeding: a randomized controlled clinical trial. BMC Womens Health. 2020;20(1):94.

75. Siegel R. The impact of self-hypnosis training on insufficient milk supply with first-time mothers: ProQuest Information & Learning; 2004.

76. Stockdale J, Sinclair M, Kernohan WG, Keller JM, Dunwoody L, Cunningham JB, et al. Feasibility study to test designer breastfeeding: a randomised controlled trial. Evidence Based Midwifery. 2008;6(3):76-82.

77. Stokes LO. Prenatal Education and Postpartum Support: Influence on Maternal Self-Efficacy and Breastfeeding Rates: The University of Arizona; 2019.

78. Stortini B, Cantin C, Croft B, Gagnier J, Fortier C, Peterson W, et al. Making an impact through an innovative youth- informed breastfeeding program for young women: Evaluating self-efficacy. Journal of Pediatric and Adolescent Gynecology. 2017;30(2):293.

79. Tseng J-F, Chen S-R, Au H-K, Chipojola R, Lee GT, Lee P-H, et al. Effectiveness of an integrated breastfeeding education program to improve self-efficacy and exclusive breastfeeding rate: A single-blind, randomised controlled study. International Journal of Nursing Studies. 2020;111:1.

80. Vincent A. The effect of breastfeeding self-efficacy on breastfeeding initiation, exclusivity, and duration: ProQuest Information & Learning; 2016.

81. Yeh C-H. Quasi-Experimental Longitudinal Cohort of the Perinatal Breastfeeding Program (PBP): Effects on Breastfeeding Outcomes in Taiwan: Case Western Reserve University; 2011.

82. You H, Lei A, Xiang J, Wang Y, Luo B, Hu J. Effects of breastfeeding education based on the self-efficacy theory on women with gestational diabetes mellitus: A CONSORT-compliant randomized controlled trial. Medicine (Baltimore). 2020;99(16):e19643.

83. Zhao Y, Lin Q, Wang J, Bao J. Effects of prenatal individualized mixed management on breastfeeding and maternal health at three days postpartum: A randomized controlled trial. Early Hum Dev. 2020;141:104944.

84. Zhao Y, Lin Q, Zhu X, Wang J. Randomized Clinical Trial of a Prenatal Breastfeeding and Mental Health Mixed Management Intervention. J Hum Lact. 2021:890334421991058.

85. Alyousefi N, Alemam A, Altwaijri D, Alarifi S, Alessa H. Predictors of Prenatal Breastfeeding Self-Efficacy in Expectant Mothers with Gestational Diabetes Mellitus. International Journal of Environmental Research & Public Health [Electronic Resource]. 2022;19(7):30.

86. Astuti Y, Sustiwi H. Family Support on Breastfeeding Self-Efficacy Among Pregnant Women. 2022. 2022;7(S2):6.

87. Avery A, Zimmermann K, Underwood PW, Magnus JH. Confident commitment is a key factor for sustained breastfeeding. Birth. 2009;36(2):141-8.

88. AYGÖR H, GEZGİNÇ K, GÜNDOĞAN KM. Breastfeeding self-efficacy in pregnant women and effective factors in the Covdi-19 pandemic. International Journal of Health Services Research and Policy.7(3):289-99.

89. Bailey J, Clark M, Shepherd R. Duration of breastfeeding in young women: psychological influences. British Journal of Midwifery. 2008;16(3):172-8.

90. Bailey J. Psychological factors influencing breastfeeding in young women [Ph.D.]. Ann Arbor: University of Surrey (United Kingdom); 2007.

91. Bartle NC, Harvey K. Explaining infant feeding: The role of previous personal and vicarious experience on attitudes, subjective norms, self-efficacy, and breastfeeding outcomes. Br J Health Psychol. 2017;22(4):763-85.

92. BAŞGÖL Ş, KÜÇÜKKAYA B. The Effect of Perceived Social Support on Prenatal Breastfeeding Self-Efficacy in Pregnants in Turkey: A web-based cross-sectional study. Kadın Sağlığı Hemşireliği Dergisi. 2022;8(3):133-43.

93. Birk DL. Self-efficacy, social support, therapeutic support and the initiation and duration of breastfeeding in adolescent mothers: University of Missouri - Saint Louis; 2005.

94. Blyth R, Creedy DK, Dennis C, Moyle W, Pratt J, De Vries SM. Effect of maternal confidence on breastfeeding duration: an application of breastfeeding self-efficacy theory. Birth: Issues in Perinatal Care. 2002;29(4):278-4.

95. Blyth RJ, Creedy DK, Dennis C, Moyle W, Pratt J, De Vries SM, et al. Breastfeeding duration in an Australian population: the influence of modifiable antenatal factors. Journal of Human Lactation. 2004;20(1):30-8.

96. Brandao S, Mendonca D, Dias CC, Pinto TM, Dennis CL, Figueiredo B. Breastfeeding Self-Efficacy in Portuguese Pregnant Women. EUROPEAN JOURNAL OF PUBLIC HEALTH. 2017;27.

97. Braun ALBS, Guedes OA, Silva PVd, Berger SB, Pedrini DL, Aranha AMF. Maternal confidence for breastfeeding and infant feeding practices in a Brazilian population. Journal of Health Sciences (Londrina). 2019;21(2):121-8.

98. Buxton KE, Gielen AC, Faden RR, Brown CH, Paige DM, Chwalow AJ. Women intending to breastfeed: predictors of early infant feeding experiences. Am J Prev Med. 1991;7(2):101-6.

99. Cabieses B, Waiblinger D, Santorelli G, McEachan RR. What factors explain pregnant women's feeding intentions in Bradford, England: a multi-methods, multi-ethnic study. BMC Pregnancy & Childbirth. 2014;14:50.

100. Chezem J, Friesen C, Boettcher J. Breastfeeding knowledge, breastfeeding confidence, and infant feeding plans: effects on actual feeding practices. JOGNN - Journal of Obstetric, Gynecologic, & Neonatal Nursing. 2003;32(1):40-7.

101. Chezem JC, Bolin J. Prenatal breastfeeding confidence is not associated with infant feeding method at one month postpartum. FASEB Journal. 2014;28(1).

102. Chong A, Biehle SN, Kooiman LY, Mickelson KD. Postnatal Depression: The Role of Breastfeeding Efficacy, Breastfeeding Duration, and Family-Work Conflict. Psychology of Women Quarterly. 2016;40(4):518.

103. Corby K, Kane D, Dayus D. Investigating Predictors of Prenatal Breastfeeding Self-Efficacy. Can J Nurs Res. 2021;53(1):56-63.

104. Craig HJ, Dietsch E. 'Too scary to think about': first time mothers' perceptions of the usefulness of antenatal breastfeeding education. Women & Birth: Journal of the Australian College of Midwives. 2010;23(4):160-5.

105. Davie P, Bick D, Chilcot J, Silverio SA. A mixed methods investigation into breastfeeding experiences of women with average birthweight babies compared to women with larger birthweight babies. Journal of Reproductive and Infant Psychology. 2022;40(2):xlii.

106. de Jager E, Broadbent J, Fuller-Tyszkiewicz M, Nagle C, McPhie S, Skouteris H. A longitudinal study of the effect of psychosocial factors on exclusive breastfeeding duration. Midwifery. 2015;31(1):103-11.

107. DemİRtaŞ HİÇYilmaz B, AÇIkgÖZ İ. The Association Between Breastfeeding Self-efficacy, Breastfeeding Attitude, Social-professional Support and Breastfeeding Control. Turkiye Klinikleri Journal of Nursing Sciences. 2017;9(2):133-43.

108. Eddy TL. Maternal psychosocial factors related to duration and exclusivity of breastfeeding practices among rural women: The healthy moms and babies study: University of Missouri-Columbia; 2015.

109. Efrat MW. Breastfeeding self-efficacy and level of acculturation among low-income pregnant Latinas. International Journal of Child Health and Nutrition. 2018;7(4):169-74.

110. Eker A, Beşen MA. The Impact of Adaptation to Pregnancy on Breastfeeding Self-Efficacy. Journal of Education & Research in Nursing / Hemsirelikte Egitim ve Arastirma Dergisi. 2021;18(2):190-7.

111. Erkal Aksoy Y, Bay H, Dereli Yilmaz S. Factors Affecting Primiparous Women's Breastfeeding Self-Efficacy Levels. Clinical Lactation. 2022;13(2):100-11.

112. Evans NT, Hsu Y-L, Sheu J-J. Path model validation of breastfeeding intention among pregnant women. Journal of Obstetric, Gynecologic, & Neonatal Nursing: Clinical Scholarship for the Care of Women, Childbearing Families, & Newborns. 2021;50(2):167-80.

113. Gijsbers B, Mesters I, Knottnerus JA, Schayck CPv. Factors associated with the initiation of breastfeeding in asthmatic families: the Attitude-Social Influence-Self-Efficacy model. Breastfeeding Medicine. 2006;1(4):236-46.

114. Gonzales AM, Jr. Breastfeeding Self-Efficacy of Early Postpartum Mothers in an Urban Municipality in the Philippines. Asian Pac Isl Nurs J. 2020;4(4):135-43.

115. Hamid SBA, Zaidi NM. Predictors of Prenatal Breastfeeding Self-Efficacy in Malaysian Women: A Cross-Sectional Study. JURNAL GIZI DAN PANGAN. 2020;15(1):53-62.

116. Herndon CH. Breastfeeding intention and initiation among rural, low-income native american and african american adolescent mothers in north carolina: Testing the theory of planned behavior: ProQuest Information & Learning; 2015.

117. Isik G, Cetisli NE. The Effect of Gestational Diabetes on Depression and Breastfeeding Self-Efficacy in Pregnancy and Postpartum Period. CLINICAL AND EXPERIMENTAL HEALTH SCIENCES. 2022;12(2):323-30.

118. Joshi A, Amadi C, Meza J, Aguirre T, Wilhelm S. Comparison of Socio-Demographic Characteristics of a Computer Based Breastfeeding Educational Intervention Among Rural Hispanic Women. J Community Health. 2015;40(5):993-1001.

119. Khresheh RM, Ahmad NM. Breastfeeding self efficacy among pregnant women in Saudi Arabia. Saudi Med J. 2018;39(11):1116-22.

120. Kools EJ, Carel T, Hein de V. The Behavioral Determinants of Breast-Feeding in the Netherlands: Predictors for the Initiation of Breast-Feeding. Health Education and Behavior. 2005;32(6):809-24.

121. Laanterä S, Pietilä A-M, Ekström A, Pölkki T. Confidence in breastfeeding among pregnant women. Western Journal of Nursing Research. 2012;34(7):933-51.

122. Lawson K, Tulloch MI. Breastfeeding duration: prenatal intentions and postnatal practices. J Adv Nurs. 1995;22(5):841-9.

123. Lemos Uchôa J, Araújo Gomes AL, Silva Joventino E, Batista Oriá MO, Barbosa Ximenes L, de Almeida PC. Sociodemographic and obstetric history in maternal self-efficacy in nursing: a study in panel. Online Brazilian Journal of Nursing. 2014;13(4):645-55.

124. Lodi JC, Milagres CS, Giovani PA, Guerra LM, Possoben RD. Impact of maternal self-efficacy and associated factors on maintaining exclusive breastfeeding in the city of Piracicaba-SP: Cohort study. MUNDO DA SAUDE. 2019;43(2):326-43.

125. Lok KY, Ko RW, Fan HS, Chau PH, Wong JY, Wang MP, et al. Feasibility and Acceptability of an Online WhatsApp Support Group on Breastfeeding: Protocol for a Randomized Controlled Trial. JMIR Res Protoc. 2022;11(3):e32338.

126. Martinez-Brockman JL, Shebl FM, Harari N, Pérez-Escamilla R. An assessment of the social cognitive predictors of exclusive breastfeeding behavior using the Health Action Process Approach. Social Science & Medicine. 2017;182:106-16.

127. McKinley E, Niroula K, Toffoli S. Assessment of Prenatal Breastfeeding Self-Efficacy and Feelings of Concern and Confusion among Pregnant Persons during COVID-19 in the United States. Journal of the Academy of Nutrition & Dietetics. 2021;121(9):A85-A.

128. McKinley EM, Knol LL, Turner LW, Burnham JJ, Graettinger KR, Hernandez-Reif M, et al. Enhancing Patient-Provider Breastfeeding Conversations: Breastfeeding Intention and Prenatal Breastfeeding Self-Efficacy among a Sample of Pregnant Women. South Med J. 2021;114(4):223-30.

129. McKinley EM. Development and validation of a self-efficacy theory-based instrument to measure prenatal breastfeeding self-efficacy and breastfeeding intention among pregnant women: ProQuest Information & Learning; 2018.

130. Minas AG, Ganga-Limando M. Social-cognitive predictors of exclusive breastfeeding among primiparous mothers in Addis Ababa, Ethiopia. PLoS ONE. 2016;11(10):e0164128.

131. Mitra AK, Khoury AJ, Hinton AW, Carothers C. Predictors of breastfeeding intention among low-income women. Matern Child Health J. 2004;8(2):65-70.

132. Mossman M, Heaman M, Dennis C, Morris M. The influence of adolescent mothers' breastfeeding confidence and attitudes on breastfeeding initiation and duration. Journal of Human Lactation. 2008;24(3):268-77.

133. Nilsson IMS, Kronborg H, Rahbek K, Strandberg-Larsen K. The significance of early breastfeeding experiences on breastfeeding self-efficacy one week postpartum. Maternal & Child Nutrition. 2020;16(3):e12986.

134. Nisa’ F, Damayanti NA, Suhariadi F, Anggraini FD, Abidah SN. The intention for providing exclusive breastfeeding associated from self-leadership and psychological capital of mother. Indian Journal of Forensic Medicine and Toxicology. 2021;15(3):2990-5.

135. Noel-Weiss J, Bassett V, Cragg B. Developing a prenatal breastfeeding workshop to support maternal breastfeeding self-efficacy. J Obstet Gynecol Neonatal Nurs. 2006;35(3):349-57.

136. O'Sullivan EJ, Alberdi G, Scully H, Kelly N, Kincaid R, Murtagh R, et al. Antenatal breastfeeding self-efficacy and breastfeeding outcomes among mothers participating in a feasibility breastfeeding-support intervention. Ir J Med Sci. 2019;188(2):569-78.

137. O'Campo P, Faden RR, Gielen AC, Wang MC. Prenatal factors associated with breastfeeding duration: recommendations for prenatal interventions. Birth. 1992;19(4):195-201.

138. Ozdemir F, Yıkar SK, Nazik E. Predictors of breastfeeding self-efficacy in pregnant adolescents. Universa Medicina. 2022;41(3):254-62.

139. Risica PM, McCausland K. Infant feeding decisions and behaviours among low-income smoke-exposed women: timing and change during pregnancy. Public Health Nutr. 2017;20(15):2796-805.

140. Robinson KM, VandeVusse L. African American women's infant feeding choices: prenatal breast-feeding self-efficacy and narratives from a black feminist perspective. J Perinat Neonatal Nurs. 2011;25(4):320-8; quiz 9-30.

141. Serrano-Alvarado K, Castro-Porras LV, Astudillo-Garcia CI, Rojas-Russell ME. Sociodemographic and Personal Predictors of Exclusive Breastfeeding in Pregnant Mexican Women Using Public Health Services. Healthcare (Basel). 2022;10(8):30.

142. Shayle A. Breastfeeding Expectations and Experiences: Associations with Mood and Well-being: University of East Anglia; 2015.

143. Shipp GM, Weatherspoon LJ, Comstock SS, Norman GS, Alexander GL, Gardiner JC, et al. Breastfeeding Self-Efficacy as a Predictor of Breastfeeding Intensity Among African American Women in the Mama Bear Feasibility Trial. Breastfeeding Medicine: The Official Journal of the Academy of Breastfeeding Medicine. 2022;17(5):453-8.

144. Stockdale J, Sinclair M, Kernohan WG. Applying the ARCS design model to breastfeeding advice by midwives in order to motivate mothers to personalise their experience. Evidence Based Midwifery. 2014;12(1):4-10.

145. Thomas JS, Yu EA, Tirmizi N, Owais A, Das SK, Rahman S, et al. Maternal knowledge, attitudes and self-efficacy in relation to intention to exclusively breastfeed among pregnant women in rural Bangladesh. Matern Child Health J. 2015;19(1):49-57.

146. Wilhelm S, Aguirre T. Feasibility of a Bilingual, Interactive, Computer-Based Breastfeeding Support Program for Rural Hispanic Women. JOGNN: Journal of Obstetric, Gynecologic & Neonatal Nursing. 2015;44:S60-S.

147. Wu SFV, Chen SC, Liu HY, Lee HL, Lin YE. Knowledge, intention, and self-efficacy associated with breastfeeding: impact of these factors on breastfeeding during postpartum hospital stays in Taiwanese women. International Journal of Environmental Research and Public Health. 2021;18(9).

148. Yazdanpanah F, Nasirzadeh M, Ahmadinia H, Abdolkarimi M. Application of the Extended Theory of Planned Behavior to Predict Exclusive Breastfeeding Intention, In Pregnant Nulliparous Women. A Cross-Sectional Study. Investigacion & Educacion en Enfermeria. 2022;40(2):47-59.

149. Yu EA, Thomas JS, Owais A, Tirmizi N, Faruque A, Das SK, et al. Maternal prenatal attitudes and postnatal breast-feeding behaviours in rural Bangladesh. Public Health Nutr. 2015;18(4):679-85.

150. Zhu J, Chan WC, Zhou X, Ye B, He HG. Predictors of breast feeding self-efficacy among Chinese mothers: a cross-sectional questionnaire survey. Midwifery. 2014;30(6):705-11.

151. Alus Tokat M, Okumus H, Dennis CL. Translation and psychometric assessment of the Breast-feeding Self-Efficacy Scale-Short Form among pregnant and postnatal women in Turkey. Midwifery. 2010;26(1):101-8.

152. Aydin A, Pasinlioglu T. Reliability and Validity of a Turkish version of the Prenatal Breastfeeding Self-Efficacy Scale. Midwifery. 2018;64:11-6.

153. Brandão S, Mendonça D, Dias CC, Pinto TM, Dennis C-L, Figueiredo B. The breastfeeding self-efficacy scale-short form: Psychometric characteristics in Portuguese pregnant women. Midwifery. 2018;66:49-55.

154. Cleveland AP, McCrone S. Development of the Breastfeeding Personal Efficacy Beliefs Inventory: a measure of women's confidence about breastfeeding. J Nurs Meas. 2005;13(2):115-27.

155. Creedy DK, Dennis C-L, Blyth R, Moyle W, Pratt J, De Vries SM. Psychometric characteristics of the Breastfeeding Self-Efficacy Scale: Data from an Australian sample. Research in Nursing & Health. 2003;26(2):143-52.

156. Dennis CL, Heaman M, Mossman M. Psychometric testing of the breastfeeding self-efficacy scale-short form among adolescents. J Adolesc Health. 2011;49(3):265-71.

157. Evcili F, Demirel G. The Prenatal Rating of Efficacy in Preparation to Breastfeed Scale (PREP to BF): A Turkish Validity and Reliability Study. CLINICAL AND EXPERIMENTAL HEALTH SCIENCES. 2020;10(3):196-202.

158. Hazar HU, Akça EU. Prenatal breastfeeding self efficacy scale: validity and reliability study. Turk Pediatri Ars. 2018;53(4):222-30.

159. Husin H, Isa Z, Ariffin R, Rahman S, Ghazi H. The Malay version of ante‑natal and postnatal breastfeeding self‑efficacy scale‑short form: reliability and validity assessment. Malaysian J Public Heal Med. 2017;17(2):62-9.

160. Iliadou M, Lykeridou K, Prezerakos P, Zyga S, Sakellari E, Vivilaki V, et al. Psychometric properties of the Greek version of the breastfeeding self-efficacy scale and correlation with depressive symptomatology. Archives of Hellenic Medicine. 2020;37(5):656-62.

161. Lau CYK, Fong DYT, Choi AYY, Ng JWY, Chu S, Tarrant M. Development and measurement properties of the Chinese breastfeeding self-regulation questionnaire. Midwifery. 2017;44:24-34.

162. McKinley EM, Knol LL, Turner LW, Burnham JJ, Graettinger KR, Hernandez-Reif M, et al. The Prenatal Rating of Efficacy in Preparation to Breastfeed Scale: A New Measurement Instrument for Prenatal Breastfeeding Self-efficacy. Journal of Human Lactation. 2019;35(1):21-31.

163. Oriá MO, Ximenes LB, de Almeida PC, Glick DF, Dennis CL. Psychometric assessment of the Brazilian version of the Breastfeeding Self-Efficacy Scale. Public Health Nurs. 2009;26(6):574-83.

164. Piñeiro-Albero RM, Ramos-Pichardo JD, Oliver-Roig A, Velandrino-Nicolás A, Richart-Martínez M, García-de-León-González R, et al. The Spanish version of the prenatal breast-feeding self-efficacy scale: reliability and validity assessment. Int J Nurs Stud. 2013;50(10):1385-90.

165. Shahry P, Vizheh M, Abbasi M, Montazeri A, Fallahian-Mehrjardi F, Dennis C-L, et al. Confirmatory Factor Analysis and Responsiveness to Change of the Persian Version of the Breastfeeding Self-Efficacy Short Form Scale. Clinical Lactation. 2021;12(3):124-36.

166. Silva-Tubio JR, Oliver-Roig A, Perpiñá-Galvañ J, Richart-Martínez M. Reliability and validity of the reduced Spanish version of the Prenatal Breastfeeding Self-Efficacy Scale. Res Nurs Health. 2021;44(6):979-91.

167. Wells KJ, Thompson NJ, Kloeblen-Tarver AS. Development and psychometric testing of the prenatal breast-feeding self-efficacy scale. Am J Health Behav. 2006;30(2):177-87.

168. Bai YK, Lee S, Overgaard K. Critical Review of Theory Use in Breastfeeding Interventions. J Hum Lact. 2019;35(3):478-500.

169. Brockway M, Benzies K, Hayden KA. Interventions to Improve Breastfeeding Self-Efficacy and Resultant Breastfeeding Rates: A Systematic Review and Meta-Analysis. J Hum Lact. 2017;33(3):486-99.

170. Chambers JA, McInnes RJ, Hoddinott P, Alder EM. A systematic review of measures assessing mothers' knowledge, attitudes, confidence and satisfaction towards breastfeeding. Breastfeed Rev. 2007;15(3):17-25.

171. Chipojola R, Chiu H-Y, Huda MH, Lin Y-M, Kuo S-Y. Effectiveness of theory-based educational interventions on breastfeeding self-efficacy and exclusive breastfeeding: A systematic review and meta-analysis. International Journal of Nursing Studies. 2020;109:103675.

172. Cummins L, Meedya S, Wilson V. Factors that positively influence in-hospital exclusive breastfeeding among women with gestational diabetes: An integrative review. Women & Birth: Journal of the Australian College of Midwives. 2022;35(1):3-10.

173. Galipeau R, Baillot A, Trottier A, Lemire L. Effectiveness of interventions on breastfeeding self-efficacy and perceived insufficient milk supply: A systematic review and meta-analysis. Matern Child Nutr. 2018;14(3):e12607.

174. Ghasemi V, Simbar M, Banaei M, Saei Ghare Naz M, Jahani Z, Nazem H. The effect of interventions on breastfeeding self-efficacy by using Bandura's theory in Iranian mothers: a systematic review. International Journal of Pediatrics. 2019;7(8):9939-54.

175. Kehinde J, O'Donnell C, Grealish A. The effectiveness of prenatal breastfeeding education on breastfeeding uptake postpartum: A systematic review. Midwifery. 2023;118:103579.

176. Maleki A, Faghihzadeh E, Youseflu S. The Effect of Educational Intervention on Improvement of Breastfeeding Self-Efficacy: A Systematic Review and Meta-Analysis. Obstet Gynecol Int. 2021;2021:5522229.

177. Morado Gonzales Jr A. Breastfeeding self-efficacy in Asia and Pacific: Scoping review. Nursing Practice Today. 2021;8(1):25-39.

178. Prasopkittikun T, Sangperm P. Self-efficacy promoting interventions for breastfeeding outcomes: an integrative review of research conducted in Thailand. Pacific Rim International Journal of Nursing Research. 2017;21(1):44-58.

179. Qian J, Wu T, Lv M, Fang Z, Chen M, Zeng Z, et al. The Value of Mobile Health in Improving Breastfeeding Outcomes Among Perinatal or Postpartum Women: Systematic Review and Meta-analysis of Randomized Controlled Trials. JMIR Mhealth Uhealth. 2021;9(7):e26098.

180. Rahmadani AN, Rahmawati AF. Meta-analysis: effect of breastfeeding education program on the breastfeeding self-efficacy and exclusive breastfeeding. Journal of Health Promotion and Behavior. 2022;7(1):42-54.

181. Seddighi A, Khalesi ZB, Majidi S. Educational Interventions to Improve Breastfeeding Self-efficacy: A Systematic Review. International Journal of Women’s Health and Reproduction Sciences. 2022;10(2):65–70.

182. Wong MS, Mou H, Chien WT. Effectiveness of educational and supportive intervention for primiparous women on breastfeeding related outcomes and breastfeeding self-efficacy: A systematic review and meta-analysis. Int J Nurs Stud. 2021;117:103874.

183. Wood NK, Woods NF, Blackburn ST, Sanders EA. Interventions that Enhance Breastfeeding Initiation, Duration, and Exclusivity: A Systematic Review. MCN Am J Matern Child Nurs. 2016;41(5):299-307.

184. Wu W, Zhang J, Silva Zolezzi I, Fries LR, Zhao A. Factors influencing breastfeeding practices in China: A meta-aggregation of qualitative studies. Maternal & Child Nutrition. 2021;17(4):e13251.
