# Supplementary material for: An exploration of prenatal breastfeeding self-efficacy: a scoping review
Source: Int J Behav Nutr Phys Act. 2024 Sep 2;21:95. doi: 10.1186/s12966-024-01641-3 (PMC11367871; doi:10.1186/s12966-024-01641-3)
Supplement: Supplementary file 2 — Supplementary Material 2 [file 12966_2024_1641_MOESM2_ESM.docx]

| **Intervention Component Category** | **Intervention Component Activity** | | **Mapping to SE Theory antecedents** |
| --- | --- | --- | --- |
| Breastfeeding Education | - Written / booklet - Pamphlet / handout / poster - Workbook / Activity sheet - Journal - Training manual - Lecture / Class - Group session - Sessions (undefined) - Workshop - Tailored information from BSES low score items - Slides / presentation / PowerPoint - Flipchart - CD / book | - Activities with cards completed with facilitator - Audio episodes - Referred to online ‘how-to’ videos / websites vetted for accurate information - Online course - Smartphone App - Hybrid App – articles, videos, quizzes, tools - Touchscreen, interactive computer package - tailored messaging - eHealth resource - Game based (quest) learning platform - Website - Web-based programme | Verbal persuasion |
| Encouragement / Support | - Counselling (in-person / online) - Advice - Encouragement - Individual / small group with LC - Availability / Access to contact details - Referral when needed - Active listening - Emotional support - Emotional / psychological support - Telephone | - Home visit - Texts / WhatsApp messages - Facebook / WhatsApp group - Peer support - Messages through interactive computer package - Links to websites - Continuity of care - Discuss normal physiological changes - Reinforce coping strategies | Verbal persuasion |

| **Intervention Component Category** | **Intervention Component Activity** | | **Mapping to SE Theory antecedents** |
| --- | --- | --- | --- |
| Engagement | - Discussion - Questions & Answers - Problem solving - Troubleshooting issues - Strategies to overcome / minimise reasons for cessation - Facilitated forum (share experience and ask Qs) - Personalised plan / tailored advice - Motivational interviewing - Assessment of previous BF experience / knowledge | - Discussing opinions, experience, difficulties and solutions - Correcting wrong knowledge - Explore previous accomplishments, incorporating skills into anticipated BF behaviour - Discuss alternate skills when negative physiological response – self-talk, problem solving, to alter perception and gain control - Normalising language (challenges not problems) | Mastery /  performance accomplishments  Verbal persuasion  Physiological and emotional states / emotional arousal |
| Vicarious / Kinaesthetic Learning | - Dolls - Models of breasts, tummy size - Model demo of positioning/latch - Practice - Technique demonstration - Return demonstration (positioning) - Breastfeeding simulation - Role play - Story of success / inspiring story - Sharing successful BF experiences | - Pregnancy suit for fathers - Groupwork - Interactive activities - Hands on problem solving - Images of breastfeeding - Video - Peer presentation - Discussion of vicarious experience with breastfeeding role models | Vicarious experience / social modelling  Mastery /  performance accomplishments |

| **Intervention Component Category** | **Intervention Component Activity** | | **Mapping to SE Theory antecedents** |
| --- | --- | --- | --- |
| Involvement / Enhancement of Social Circle | - Encourage to attend local support group - Information on local support groups - Develop social support - Consideration of social support - Texts to partner / family - Enhancing social support - Partner / family member involvement in education - Getting support | - ‘Father/partner as co-parent’ module - Father-focused antenatal BF class - Opportunity for significant persons to learn how to support and encourage - Smartphone app for fathers - Resources in local area - Encouragement of partner to support and assist - Assessment of previous family / partner support | Physiological and emotional states / emotional arousal  Verbal persuasion |
