# Supplementary figures and images for: An exploration of prenatal breastfeeding self-efficacy: a scoping review
Source: Int J Behav Nutr Phys Act. 2024 Sep 2;21:95. doi: 10.1186/s12966-024-01641-3 (PMC11367871; doi:10.1186/s12966-024-01641-3)

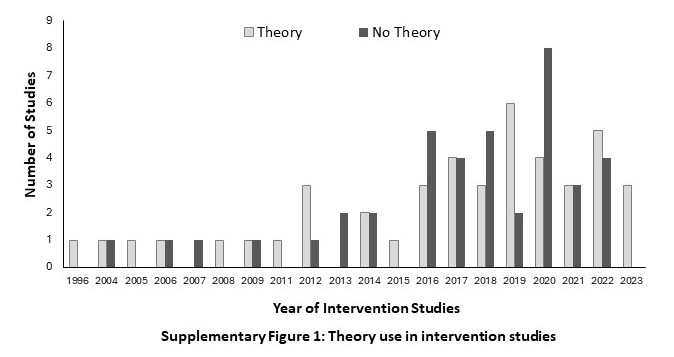

Supplement: Supplementary file 3 — Supplementary Material 3 [file 12966_2024_1641_MOESM3_ESM.jpg]
